# Next-Generation Green Hydrogen: Progress and Perspective from Electricity, Catalyst to Electrolyte in Electrocatalytic Water Splitting

**DOI:** 10.1007/s40820-024-01424-2

**Published:** 2024-07-05

**Authors:** Xueqing Gao, Yutong Chen, Yujun Wang, Luyao Zhao, Xingyuan Zhao, Juan Du, Haixia Wu, Aibing Chen

**Affiliations:** College of Chemical and Pharmaceutical Engineering, Hebei University of Science and Technology, Shijiazhuang, 050018 People’s Republic of China

**Keywords:** Hydrogen, Electrolysis, Hydrogen production, Renewable energy, Catalyst

## Abstract

Green hydrogen from electrolysis of water has attracted widespread attention as a renewable power source. Among several hydrogen production methods, it has become the most promising technology. However, there is no large-scale renewable hydrogen production system currently that can compete with conventional fossil fuel hydrogen production. Renewable energy electrocatalytic water splitting is an ideal production technology with environmental cleanliness protection and good hydrogen purity, which meet the requirements of future development. This review summarizes and introduces the current status of hydrogen production by water splitting from three aspects: electricity, catalyst and electrolyte. In particular, the present situation and the latest progress of the key sources of power, catalytic materials and electrolyzers for electrocatalytic water splitting are introduced. Finally, the problems of hydrogen generation from electrolytic water splitting and directions of next-generation green hydrogen in the future are discussed and outlooked. It is expected that this review will have an important impact on the field of hydrogen production from water.

**Graphical Abstract:**

## Highlights

- This review systematically summarizes the source of electricity, the key choice of catalyst, and the potentiality of electrolyte for prospective hydrogen generation.
- Each section provides comprehensive overview, detailed comparison and obvious advantages in these system configurations.
- The problems of hydrogen generation from electrolytic water splitting and directions of next-generation green hydrogen in the future are discussed and outlooked.

## Graphical abstract

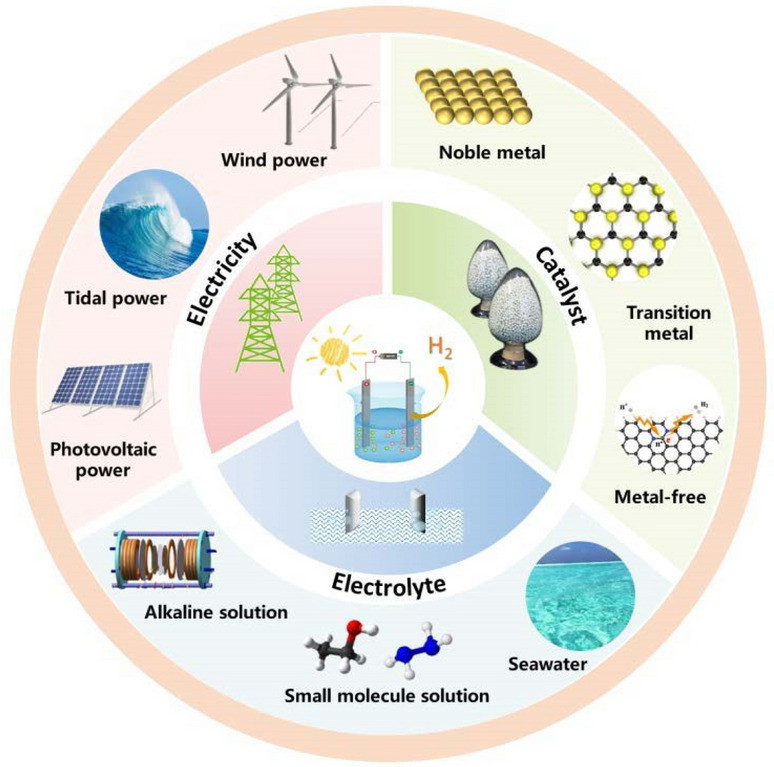

## Introduction

Hydrogen, a renewable and clean power source, has an important place in the future, and its preparation, storage, transport and application have attracted much attention [1, 2]. Now, the main technical means of hydrogen production include hydrogen production by fossil energy reforming, hydrogen manufacturing from industrial by-product gas and hydrogen generation through electrolysis of water [3]. Traditional fossil fuel hydrogen production technology is mature, but fossil fuel resources are limited. When burned, it will cause carbon emissions and seriously pollute the environment [4]. Industrial by-production of hydrogen refers to the technology of extracting hydrogen from coke oven gas, chlor-alkali tail gas and other by-products generated during industrial production. Due to technological limitations, the hydrogen produced by this method is of low purity, and there are still pollution problems in the production process [5].

Hydrogen production by electrolysis is a green and efficient hydrogen production technology based on the principle of electrodes splitting water molecules into hydrogen and oxygen using electricity [6]. There are many technologies of hydrogen production through electrolysis of water, such as high temperature solid oxides, proton exchange membrane, alkaline water and anion exchange membrane electrolysis [7–10]. Hydrogen production by water electrolysis is of high purity and is a good choice to solve the problem of retained renewable energy. The whole process of hydrogen production by electrolysis of water only consumes water and electricity, and it does not consume other fossil resources. The process is simple and easy to operate, and the product is carbon-free clean and non-polluting. The equipment occupies a small area, and it can be used to produce more than one piece of equipment at the same time with flexible operation. But at the same time water electrolysis for produce hydrogen is also a kind of expensive hydrogen technology. The main power consumption of electricity to produce hydrogen is about 4.5–5.5 kW h m^−3^ [11, 12].

**Fig. 1:**
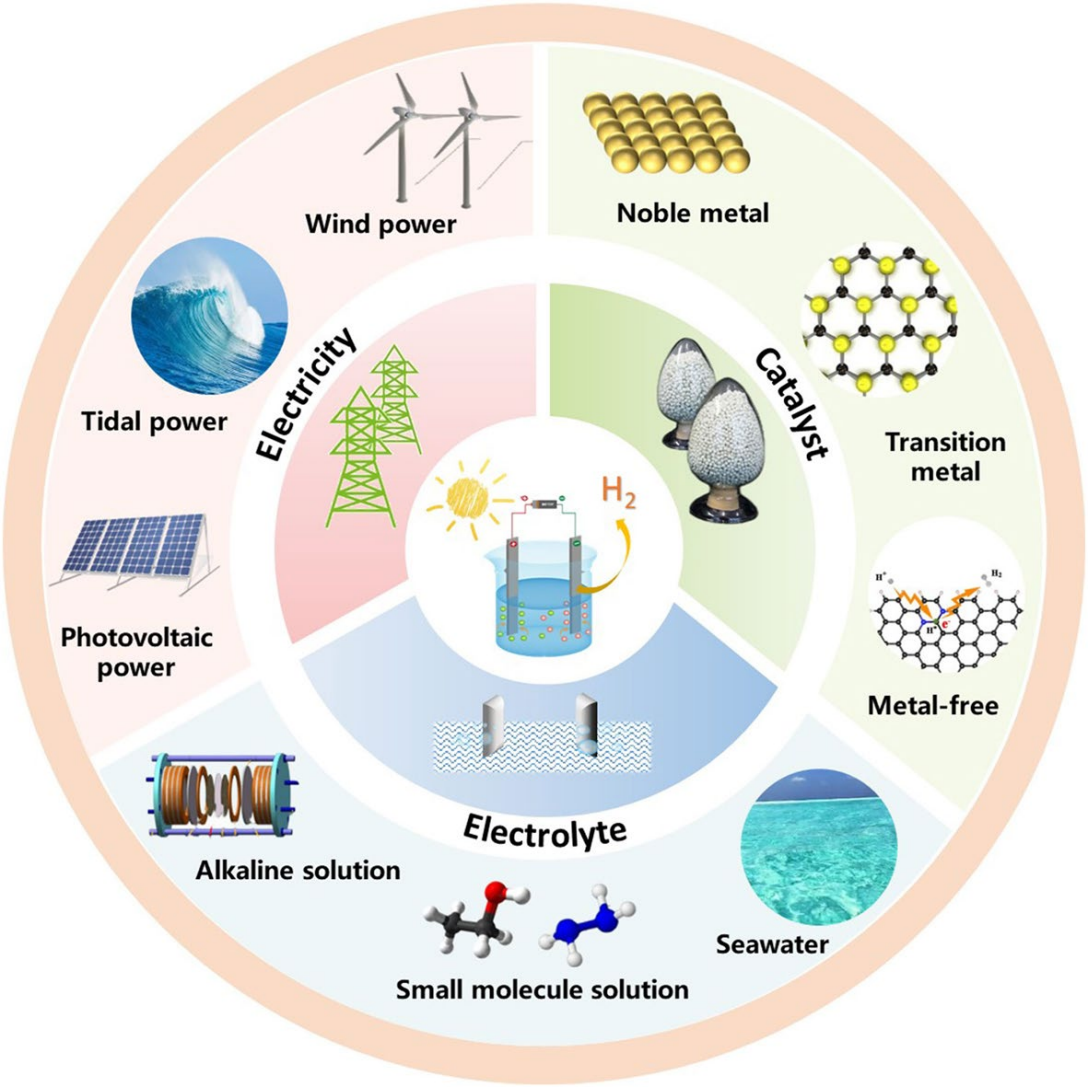
Schematic diagram for green hydrogen production by water splitting from electricity, catalyst and electrolyte. Reproduced with permission [13]. Copyright 2019, John Wiley and Sons Ltd

Here, this paper systematically summarizes from the source of electricity, the key choice of catalyst and the potentiality of electrolyte (Fig. 1) and puts forward the prospect of the new technical development direction of water electrolysis to hydrogen production. Firstly from an economic point of view, we will start with an introduction to the source of electricity, explaining the choice of suitable power supply energy and the design of efficient power supply configuration. Then, we mainly summarized the progress of the current catalysts for hydrogen production by electrolysis of water, which are divided into three categories: precious metals, transition metals and metal-free materials. In addition, we will explore the impact of different electrolyte choices on energy efficiency, including alkaline solution, seawater and small molecule electrolysis systems. Each segment offers a thorough examination and overview of the weakness and apparent benefits inherent in these system configurations, featuring lucid and easily understandable comparisons. Conclusively, we delve into the obstacles encountered, offering insights into the future trajectory of development for these electrocatalytic systems. The overarching aim of crafting this review is to present a current status report on the recent strides made in effective electrocatalytic systems, emphasizing the promising prospects of these advanced technologies for forthcoming green hydrogen production.

## Power Supply for Water Electrolysis

At the moment, environmental problems and energy consumption are gradually exposed to humans. In these cases, the use of clean energy is gradually entering the right track. Among numerous energy sources, hydrogen power has received special attention. Obtaining hydrogen energy through electrolysis of water can greatly alleviate energy scarcity and pollution problems. In order to fully leverage the advantages of hydrogen energy, its production process is very important. In general, hydrogen gas production requires electrolysis, and power sources is crucial. If the most traditional thermal power generation is adopted, the environmental pollution caused by its process will greatly discount the achievements of clean energy production. To address this issue, we can adopt relatively environmentally friendly power generation methods to provide sufficient power resources for equipment, such as wind hydrogen coupling, tidal, photovoltaic and other environmentally friendly power generation modes [14].

### Wind Power Generation

The global hydrogen power industry is developing rapidly, and the coupling of hydrogen power and new energy generation has attracted attention. Each region develops coupled hydrogen production from abundant resources according to its own resource advantages, which can effectively reduce the cost of hydrogen production [15–22]. In areas with abundant ocean and wind resources, the use of electrolysis of wind hydrogen coupling to electrolysis seawater has become a method to reduce the cost of hydrogen production. Some people have designed a hydrogen network model based on wind power grid wind–hydrogen coupling, and an optimal regulation method to increase the operational efficiency has been proposed [23–25]. The simulation results of Qinghai wind farm show that the proposed method improves the reliability of the wind–hydrogen coupling power generation system. Fang et al. established an optimization model of wind and hydrogen coupled power generation using wind and hydrogen energy technology to maintain the stability of wind power and the return on investment of enterprises [26]. They calculated the fluctuating cost of wind energy to obtain the maximum capacity of each generating unit. Schmidt et al. developed an energy management control strategy that considers the impact of wind energy fluctuations on the operating performance of electrolytic batteries [27]. Research has shown that this energy management control strategy can shorten battery conversion time by 93.5%, which can increase the system's hydrogen production. Hing et al. developed a new development plan to systematically develop wind energy [28]. Sharma et al. calculated that wind speeds of 4 m s^−1^ or higher are suitable for the utilization of wind energy [29]. Gallagher and his colleagues reported a system using offshore wind farms as power sources [30]. Crivellari and Cozzani proposed a significant strategy to convert offshore wind power into chemical energy, rather than directly utilizing electricity [31].

At present, offshore wind power is in a rapid development stage. The wind hydrogen coupled power generation system has attracted the attention of academic and technical experts at home and abroad. However, there are also some inevitable problems, such as security issues [32, 33]. Besides, the capacity utilization rate is not high because of the randomness of the wind resource, the size of the wind power and the time of generation cannot be determined. So there are still many problems to be solved in the process of promoting its practical application.

### Tidal Power Generation

Tidal energy storage capacity is large, and many countries have invested relatively in tidal power generation technology. There is no serious pollution at route of using tidal energy for power manufacture. Establishing tidal power stations in seaports and bays can avoid occupying civilian farmland [34]. Scholars around the world have conducted extensive research in this field and made significant progress. The principle of tidal power stations is to use tidal power generation, which is essentially artificial water storage. The stored water can be used to build dams or lagoons. In China, tidal energy was first used in the early eleventh century. With the development of technology, it was not used for power generation until the 1950s. At present, China's tidal power generation units have been vigorously developed [35]. According to rough statistics, eight tidal power stations with an installed capacity of 6000 kW can operate normally and generate electricity, with an average annual power generation of 10,000 kW h^−1^. Zhejiang Jiangxia Tidal Experimental Power Station adopts the form of bidirectional ball bubble water turbine generator units to achieve bidirectional power generation. It is equipped with 6 units, with a single unit capacity of 500–700 kW. The last generating unit of the power station was retrofitted by Longyuan Power Generation Company. The prefabricated power station has been expanded to 4100 kW, generating nearly 800 × 10 kW h^−1^ per day, achieving commercialization preliminarily. In February 2022, the largest single unit tidal power generating unit in Zhoushan, Zhejiang province bagan to construct. The Endeavor unit is the fourth generation of megawatt level tidal power generation units, with a self-weight of 325 tons and a single unit capacity of 1.6 megawatts, which is five times higher than the previous generation. In recent years, the total annual power generation of tidal power stations worldwide has grown rapidly. It is expected to reach 60 billion kW h^−1^ by 2030. In recent years, people's understanding of the potential for tidal energy development has steadily increased, with projects being constructed in the USA, Canada, the UK and India. In July 2021, the world's largest tidal power generation unit was put into operation in the waters off the UK, attracting attention in this field. The Atlantis is a tile tidal turbine, which belongs to the horizontal axis tidal current turbine [36]. In addition, Yuan et al. studied a horizontal axis tidal current turbine with reverse rotation; they conducted a detailed study on the impact of relevant parameters on energy collection efficiency and verified the reliability of their design [37].

Tidal energy is a promising renewable marine energy source. Tidal energy is mainly composed of water level potential energy and kinetic energy carried by ocean currents, which are divided into rising tide and falling tide. However, there are technical barriers for hydrogen production by tidal power devices due to issues such as the high cost of tidal energy and its low rate of practical application.

### Photovoltaic Power Generation

Photovoltaic (PV) power generation is one of the indispensable pollution-free power generation methods in the twenty-first century, which has entered people's daily life from the beginning. Since the proposal of the 14th Five Year Plan, the proportion of photovoltaic has been increasing, and China's photovoltaic industry has shown strong development momentum. In order to find the optimal location and size for photovoltaic landing, people need to deepen their understanding of the characteristics of traditional distribution networks. By of 2020, eight PV power plants have been built nationwide, and the total power generation and investment costs are developing in a more favorable direction. PV poverty alleviation projects are being vigorously promoted nationwide, and the structural power grid of rural areas affected by PV poverty alleviation is very fragile. It is urgent to improve the ability of PV absorption of these areas [37–39]. The use of PV as a power source to green hydrogen through water splitting, which may become one of the main ways of clean energy production in the next few years [40, 41]. The "Smart Photovoltaic Industry Innovation and Development Action Plan" issued in 2022 proposes to grasp the development trend of the digital economy, promote the continuous development of PV power generation industry of China, and promote PV industry to move toward refinement. Accordingly, photovoltaic power generation has received many policy support and development opportunities. Xu et al. searched for the development direction of PV industry in China and constructed a systematic energy environment and economic model of PV industry [42]. Zhou et al. hold an optimistic attitude toward the future development of Chinese PV and to guide the healthy development of the PV power market [43].

Although PV industry has been at the forefront, there are still some issues that need to be improved and resolved. PV power generation relies on the instability of solar radiation weather conditions, such as cloudy days, rainy seasons or short sunshine hours in winter, which may lead to a reduction in PV power generation, all of which may have an impact on power generation capacity. In the future, we should strengthen the breakthroughs cutting-edge power generation technologies and reinforce the layout and energy storage capacity of the grid. Solar energy resources should be fully utilized, combined with electrolytic water hydrogen technology, to produce cleaner and more convenient hydrogen energy.

### Economic Analysis of Power Sources for Hydrogen Production

The electricity accounted for about 80% of total cost of hydrogen production. Therefore, the combination of electrolytic water hydrogen production technology with efficient, economical and pollution-free renewable energy generation technology has great development and application space. Wind power, tidal energy and PV power are all important ways to effectively utilize renewable natural resources. Wind power generation technology has become increasingly mature. The energy efficiency has reached more than 95%; the cost of power generation is also relatively low. If we consider the environmental pollution of coal power costs and transport and other investments, wind power costs are lower than coal power. But from the policy point of view, the state encourages wind power generation, there are large-scale financial subsidies, but the actual situation is not ideal. Tidal energy is a new kind of environmental protection marine energy. It does not cause any harm to the surrounding environment, but also can reduce CO_2_, SO_*x*_, NO_*x*_, dust emissions. Due to the construction and operation of tidal power stations, there will be adverse effects on the ecology and economy of the surrounding areas. As a result, operation and maintenance costs will increase. A series of tax incentives to encourage the development of PV power generation has been provided, such as tax reductions and tax credits. However, PV power generation requires a large amount of land for the layout of PV modules, which may have an impact on local land use and ecology, which may increase costs. Although photovoltaic power generation is a clean energy source, some environmental pollution and waste may be generated during the manufacturing and processing of photovoltaic modules, which requires additional manpower and material resources to properly treat and manage these polluting wastes, which also means an increase in costs.

### Comparison of Three Renewable Electricity Sources

**Fig. 2:**
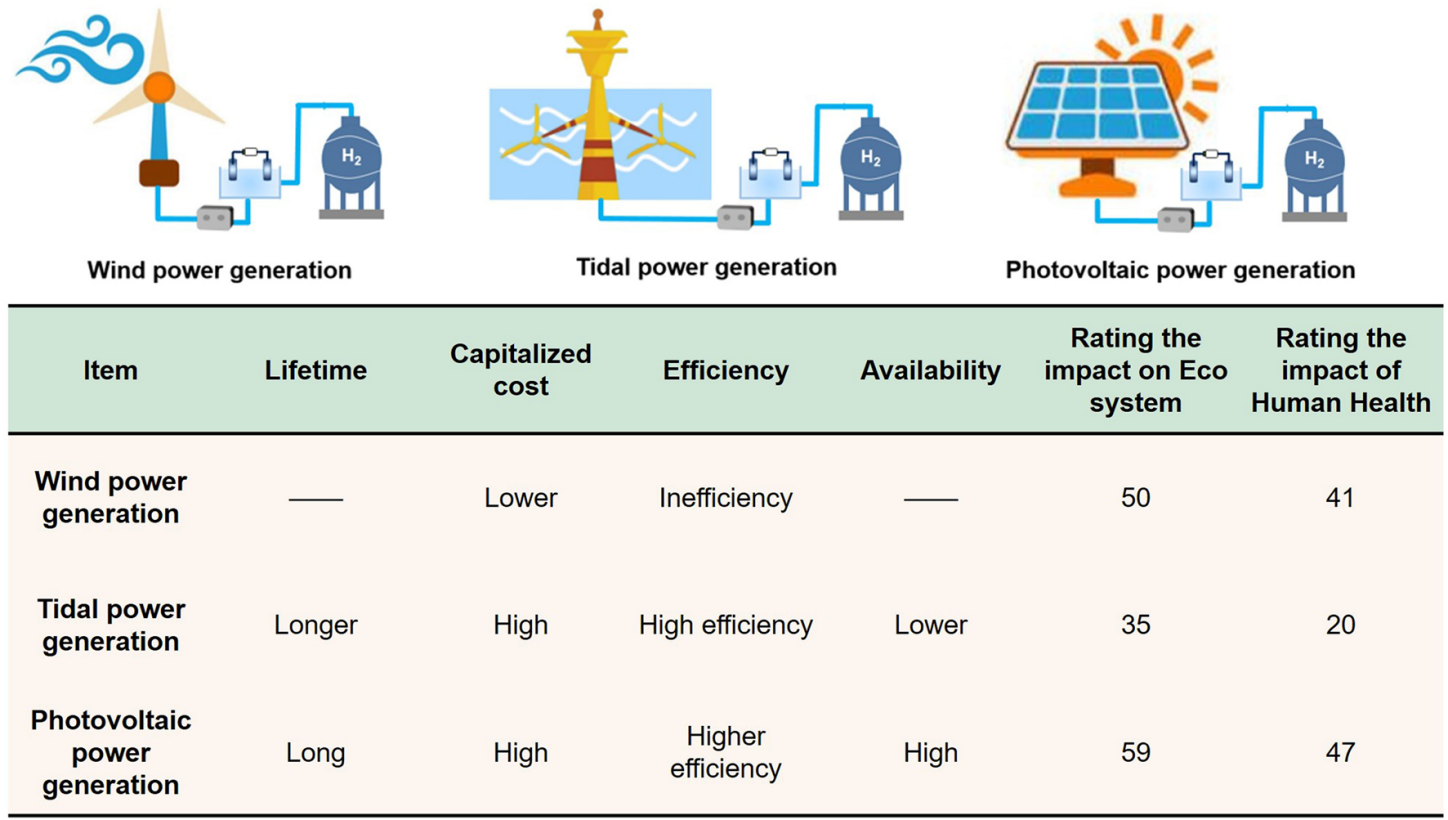
Comparison of parameters of renewable energy generation (The values represent the environmental impact scores of human health and ecosystems; the higher the value, the greater the harm to human health/ecosystem, which will affect the public health and damage the environment [45])

Electricity production is an industry with high energy consumption and demand. To this end, we will actively develop new energy and replace non-renewable resources with renewable energy. Common new energy sources include natural resources such as tidal energy, solar energy, ocean energy, geothermal energy and wind energy. Therefore, in order to meet human energy needs, relevant industries around the world are increasing their efforts to develop cleaner and more efficient power generation technologies. Among them, wind energy and solar energy have made significant progress in power generation, reducing the use of fossil fuels. But with continuous increase in frequency of new energy use, there are also some problems that need to be solved, such as climate impact, high pollution and high energy consumption [44]. In addition, it is expected that by 2030, discarded photovoltaic modules will generate up to 2000 tons of waste. Therefore, we must improve the recycling measures for solar power generation equipment, reduce the raw material cost of solar cells, extend the service life of photovoltaic products, while reducing the impact on the environment. The parameters of several power generation technologies are summarized (Fig. 2). The use of clean and renewable electricity can fully leverage the value advantages of electrolytic water hydrogen production equipment.

## Catalysts for Water Electrolysis

The problem of half-reaction, hydrogen and oxygen evolution reactions is that their kinetics are slow, resulting in a relatively low energy conversion efficiency [46–49]. Noble metal catalysts with excellent water electrolysis performance can improve the efficiency by improving the reaction kinetics [50–56]. However, due to their high price and scarce reserves, it is difficult to support large-scale production and application [57]. Therefore, research on electrocatalysts has mainly focused on the development and use of various catalysts with low noble metal content and non-noble metal catalysts, such as finding high-performance transition metal catalysts and metal-free catalysts [47, 58–62].

### Overview of Water Electrolysis

#### Hydrogen Evolution Reaction

The hydrogen released reaction is normally referred to as hydrogen evolution reaction (HER). In the process of hydrogen production from electrolytic water, HER is a multi-step two-electron transfer reaction, and water or H^+^ is reduced to H_2_ at the cathode [63].

#### Oxygen Evolution Reaction

The reaction in which oxygen is released is often mentioned as oxygen evolution reaction (OER). OER involves four electrons; water or OH^−^ is oxidized at the anode to form O_2_ and H_2_O. The kinetics of the reaction is slow and therefore requires a high overpotential.

#### Total Water Splitting

Full electrolysis of water refers to the simultaneous occurrence of HER and OER half-reactions in the same electrolyte. The main energy consumption of the electrolyzer is to overcome the slow kinetics of HER and OER [64], so it is necessary to use catalysts with high energy conversion efficiency to reduce the overpotential of the electrode reaction.

### Performance Evaluation Parameters of Electrocatalysts

In order to scientifically evaluate the HER and OER activities of different electrocatalysts, many important parameters such as overpotential, Tafel slope, electrochemical active area, electrochemical impedance and stability are introduced.

#### Overpotential

In addition to actual chemical requirements to drive reaction, the extra potential is known as overpotential (*η*). It is a fundamental parameter to measure the reactivity of electrocatalysts toward water splitting. At the same current density, the smaller the overpotential, the higher activity of the chemical agent [65].

#### Tafel Slope

The Tafel relationship between overpotential (*η*) and current density (*j*) is satisfied by the equation *η* = *a* + *b*log*j*. where *b* is the Tafel slope. The Tafel plot to overpotential is a tool to quantify the extent of reaction kinetics in electrocatalytic processes. The slope of the Tafel plot explains the potential of mV dec^−1^ required to drive the reaction. The faster the reaction kinetics, the lower the Tafel slope and vice versa. Therefore, a low Tafel slope is an important factor in the analysis of effective electrocatalysts in electrochemical reactions [66, 67].

#### Electrochemical Active Area

The electrochemically active area (ECSA) reflects the ability of the catalyst to adsorb or desorb water molecules and gaseous products, as well as the number of active sites. The value of ECSA is directly proportional to the catalyst's double-layer capacitance (*C*_dl_), which is expressed as ECSA = *C*_dl_/*C*_s_, where *C*_s_ is the specific capacitance of the corresponding surface-smoothed sample under the same conditions. The double-layer capacitance can be calculated by a non-Faradaic region of the cyclic voltammetry (CV) curve calculated by plotting Δ*j* versus scan rate Δ*v* for a given potential; the slope of the resulting straight line is twice the value of *C*_dl_. Larger *C*_dl_ values indicate higher exposure of the active site and a larger ECSA from the surface [68].

#### Stability

Stability is a key parameter to evaluate whether electrolytic water catalysts can be used in practical applications. Constant potential and current tests are carried out to evaluate the long-term stability of the electrode materials, which is usually assessed by the chronoamperometry and chronopotentiometry methods [69]. Timed current method refers to setting a certain overpotential and recording the change of current density with time. Timed potential refers to a constant current density during electrolysis, and the change of potential with time is recorded. The smaller the change in the current density or potential before and after, the longer the duration, the more stable the catalyst performance [70].

### Electrocatalytic Materials for Water Electrolysis

#### Noble Metal Catalyst

Many experimental data and theories have shown that Pt metal has suitable adsorption energy for H_ads_ in the electrolytic water catalytic reaction, making it the most commonly used HER catalyst for industrial electrolyzed water [71]. Noble metal Pt-based catalysts have low overpotential and high exchange current density, which can effectively reduce the hydrolysis voltage. Liu et al. reported a platinum-copper nanosphere catalyst with a three-component heterostructure. This catalyst outperforms some state-of-the-art platinum monoatomic catalysts [72].

**Fig. 3:**
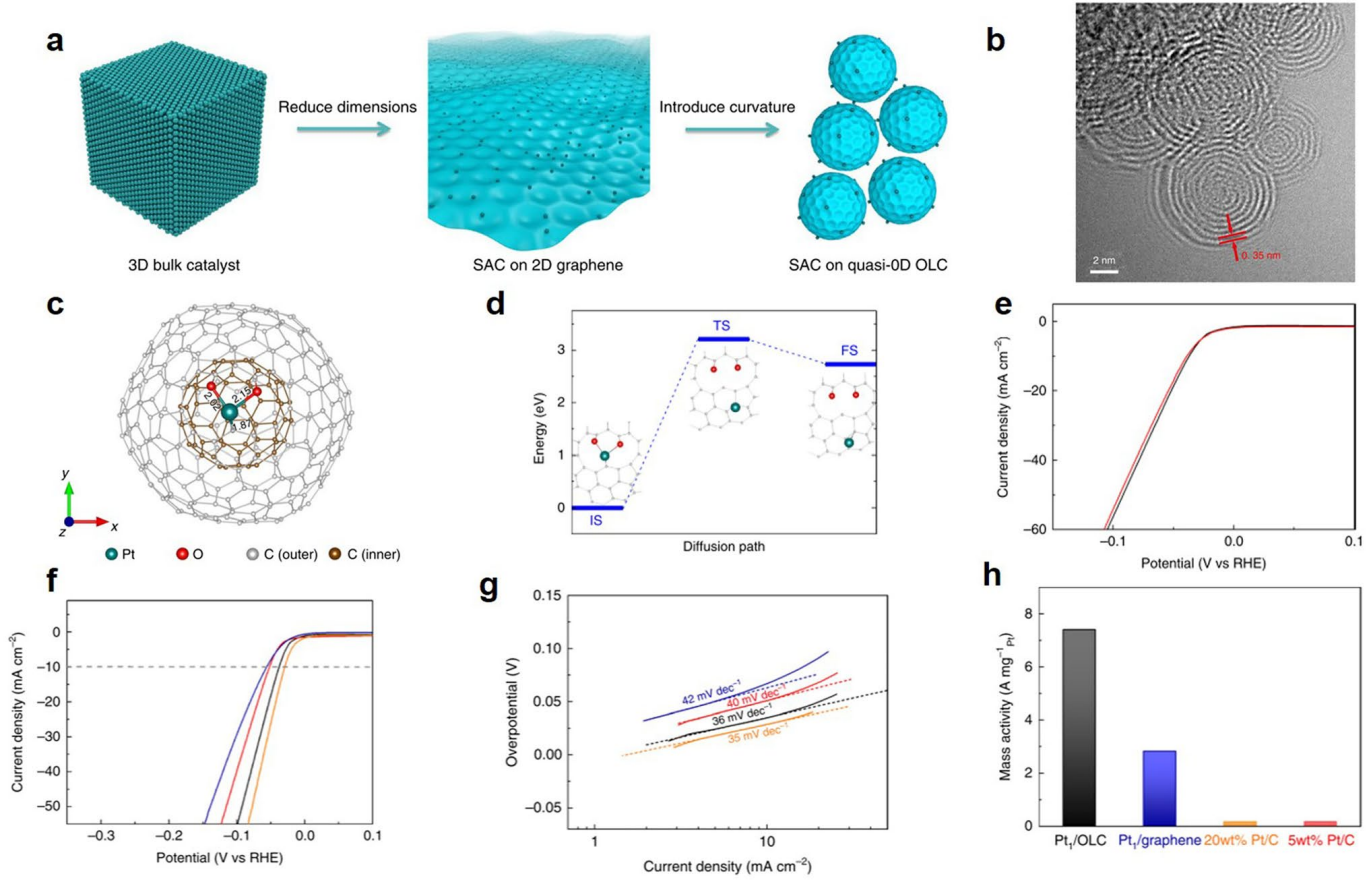
**a** PT/OLC catalyst preparation schematic diagram. **b** TEM image of Pt_1_/OLC showed the polyshell fullerene structure. **c** The optimized PtO_2_C_295_ atom model. **d** The lowest diffusion barrier of Pt atoms on PtO_2_C_295_. **e** Polarization curves for acceleration stability measurements in 0.5 M H_2_SO_4_ electrolyte. **f** LSV curves of different samples in 0.5 M H_2_SO_4_ electrolyte. **g** Tafel plots. **h** The mass activity. Reproduced with permission [85]. Copyright 2019, Springer Nature

Although it is the most commonly used HER catalyst, its high price and poor resource storage limit its large-scale industrial application. Currently, noble metal catalyst research is focused on adjusting their structure and composition [73]. Core–shell noble metal catalyst reduces the amount of noble metal used while enhancing catalytic performance because of the synergistic effect with the core metal [74, 75]. Addition of a small amount of transition metal or nonmetal atoms to the noble metal catalyst can improve the catalytic activity while reducing the application cost, which is currently being actively sought [76, 77]. Liu et al. prepared multilayer RuNi alloy nanosheets with good alkaline solution properties [78]. Yan et al. used IrCl_3_ instead of the pre-synthesized Ni network to obtain a three-dimensional porous NiIr alloy catalyst. In alkaline electrolyte, overpotential of 22 mV at 10 mA cm^−2^ [79]. Wu et al. used Co and Ni as doping elements to construct Co and Ni co-doped RuO_2_ catalytic material, which effectively reduced the amount of precious metal elements. Meanwhile, OER activity of RuO_2_ catalysts was improved by adjusting the doping amounts of Co and Ni elements [80]. Chen optimized d-band center of Ru site in RuO_2_ material by using Mn element doping, which optimized adsorption energy of its surface for OER intermediates. OER activity was substantially improved compared with that of pure RuO_2_ catalysts [81]. Ying et al. prepared co-elemental doped IrO_2_ two-dimensional nanoframeworks catalytic materials, which had significantly higher OER activity than IrO_2_ [82]. In addition, Joshi prepared B-doped IrO_2_ composite reducing graphene oxide catalytic materials by a one-step polymerization reaction, doped with only 2% B elements, but reduced the overpotential of the OER reaction by about 100 mV compared to pure IrO_2_ nanoparticle materials [83]. Zhang et al. and their research group [84] investigated the conjugation effect between electron donor B and Ir and prepared N, B co-doped Ir@NBD−C. This catalyst exhibited significant anti-HER activity at an ultra-low overpotential of 7 mV (10 mA cm^−2^), which was superior to almost all HER electrocatalysts. Characterization and theoretical calculations show that the outstanding catalytic activity can be attributed to the optimal binding of a hydrogen intermediate species (H^*^) with an adjustable and favorable electronic structure from the Ir site through the binding of B heteroatoms. Song and Jiang et al. prepared Pt/OLC catalysts by anchoring Pt atomic particles to onion-like carbon nanospheres (OLECs) [85]. Since this high curvature structure of the multichiral fullerene effectively enhances the activity of platinum (Fig. 3a, b), they modeled PtO_2_C_295_ by encapsulating C_60_ in a fullerene lacking C_235_, where the platinum atoms are bonded to one C and two O atoms on surface (Fig. 3c). The diffusion of Pt on the surface of PtO_2_C_295_ required to overcome a high energy barrier of 3.20 eV, indicating excellent structural stability of this arrangement (Fig. 3d). The Pt metal-based catalyst has a negligible initial potential near the thermodynamic potential of HER, and the catalyst has a Tafel slope of 20 wt% Pt/C, which is a superior performance. Figure 3e−h shows that the current density has the same as the initial curve and the potential changes only slightly, indicating the durability of the Pt_1_/OLC catalyst. Zhang et al. used N-doped carbon nanotubes (CNTs) as carriers for Pt deposition and found that nitrogen-rich carriers were beneficial to the deposition of Pt single atoms [86]. Its performance was superior compared to that of commercial Pt/C catalysts. Except carbon support, many studies have successfully synthesized single atom catalysts using materials such as MoS_2_, FeO_x_, TiN as carriers, effectively reducing the loading of Pt [87, 88].

The noble metal ruthenium (Ru) also has good electrochemical performance and is cheaper than Pt. Silicon carbide-supported Ru is a good hydrogen production catalyst, and the pores of silicon carbide make the temperature distribution inside the catalyst uniform, making it suitable for catalytic reactions [89]. However, its stability is poor and it is prone to agglomeration. It can be solved by alloying, element doping and anchoring [90–92]. Wu et al. prepared three-dimensional nano-porous CuRu alloy by alloying method and calculated the HER activation energy using density functional theory [93]. Compared with pure Cu or pure Ru, this alloy effectively reduced the hydrolysis ionization energy barrier and optimized hydrogen adsorption desorption energy. The HER performance of the catalyst in alkaline/neutral electrolytes was significantly improved.

**Fig. 4:**
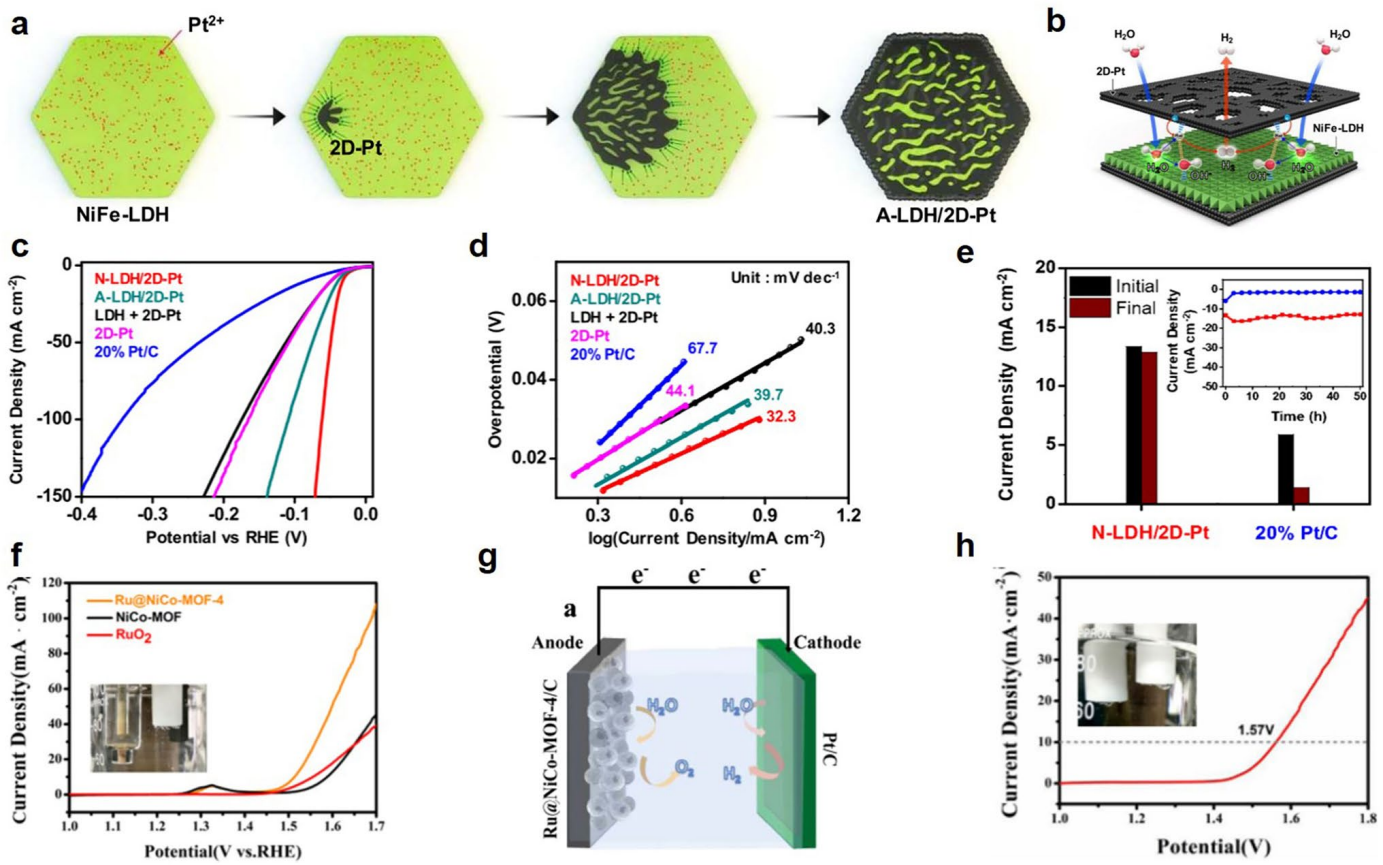
**a** Schematic diagram of NiFe−LDH/2D−Pt preparation. **b** Hydrogen production mechanism of NiFe−LDH/2D−Pt. **c** LSV curves of HER performance. **d** Tafel plots. **e** CA curves for the two catalysts measured. Reproduced with permission [94]. Copyright 2020, American Chemical Society. **f** LSV curves of OER. **g** Schematic diagram of the overall hydrolysis. **h** LSV curves of alkaline electrolyzer. Reproduced with permission [96]. Copyright 2021, American Chemical Society

The combination of precious metals and non-precious metals can not only reduce the content of precious metals, but also significantly improve the catalytic activity. Jang et al. prepared a Pt catalyst with a 2D structure using NiFe−LDH as a template (Fig. 4a, b) [94]. The catalyst exhibited excellent HER performance with approximately sixfold increase in specific activity compared to the 20% Pt/C. In addition, the catalyst exhibited a nearly constant chronoelectric current curve with good stability (Fig. 4c−e). Niu et al. prepared a RuO_2_/(Co,Mn)_3_O_4_ nanocomposite catalyst, which effectively reduced the amount of precious metal Ru [95]. The mass ratio of Ru in this catalyst was only 2.51%, but the catalyst has excellent OER activity, which is significantly higher than that of the single RuO_2_ catalyst. Liu et al. added Ru as a doping element to NiCo−MOF porous nanospheres [96]. Due to doping of Ru, the intrinsic activity of the NiCo−MOF catalytic material is greatly enhanced, and the oxygen evolution performance is improved, reaching a 284 mV overpotential at a current density of 10, while mass specific activity of catalyst reaches 310 mA mg^−1^ (Fig. 4f). Because of the extraordinary OER performance of Ru@NiCo-MOF-4, we further performed global water splitting tests in alkaline solution using a two-electrode system (Fig. 4g). Figure 4h shows the performance of a two-electrode alkaline electrolyzer, which has an excellent electrolyzer performance with a potential up to 1.56 V at 10 mA cm^−2^. Li et al. used carboxylic acid graphite nanosheets to anchor Ru^3+^ and uniformly loaded Ru nanoparticles after annealing and reduction. Its activity conditions are equivalent to commercial Pt/C catalysts in acid [97], while there are numerous methods to enhance the efficiency of metal catalysts. But relying on noble metals for extensive hydrogen production is not a sustainable solution. Therefore, it is imperative to intensify research on non-noble metal catalysts, including transition metal and nonmetal catalysts.

#### Transition Metal Catalyst

Transition metals have outstanding electrocatalytic HER and OER activities. They are inexpensive and abundant, making them ideal alternatives to replace noble metal catalysts. Transition metals and their alloys, oxides [98–100], sulfides [101], nitrides [102–104] and phosphides [105, 106] have excellent stability and reactivity. The performance of certain HER and OER catalysts [65, 107–115] is comparable to that of noble metal catalysts, making them suitable for industrial-scale hydrogen energy production.

In order to improve the performance of transition metal catalysts, many strategies have been developed. In terms of active site regulation, synergistic interaction of dual active sites can enable the catalysts to achieve superior performance. Mu et al. and their group [116] obtained highly efficient N-doped carbon nanotube-encapsulated Co_2_P−CoN dual active center electrocatalyst with trifunctional performance for catalyzing of HER, OER and ORR. Yuan et al. and their group [117] designed and fabricated nanoscale hybrid Mo_2_C−CoO (Mo_2_C−CoO@N−CNFs) encapsulated in N-doped carbon nanofibers. Due to the synergistic effect of bimetallic Mo/Co, the kinetics of HER is accelerated and the energy barrier of OER is reduced. Thus, the catalyst showed significant catalytic activity in both OER and HER. In terms of structural regulation, catalysts with specific structure such as three-dimensional and array structure can effectively improve the catalytic performance of the catalyst. Patolsky et al. and their group [118] synthesized electronic structure engineering 3D layered nanostructures of highly conductive NiS_x_ electrocatalysts. The three-dimensional structure makes the sample have excellent hydrogen evolution and oxygen evolution properties. Huang et al. and their group [119] prepared a nanoparticle array of Mn-doped nickel−cobalt phosphide (Mn−NiCoP). Due to the synergistic effect of the optimal amount of doping and array structure, the OER and HER performances are improved simultaneously. At a high current density of 100 mA cm^−2^, an overpotential of only 148 mV was required for HER and 266 mV for OER.

Adsorption energy of key intermediates, d-band center structure of the catalyst, can be significantly adjusted and affected by heterojunction forming and ion etching strategies. Xie et al. proposed an Ni_2_P/FeP_2_ heterostructure to form an internal polarization field (IPF) that causes hydroxyl overflow (HOSo) during OER [120]. The orientation transition of HOSo from FeP_2_ to Ni_2_P facilitated by IPF can activate the Ni site, form a new hydroxyl transfer channel and establish an optimized reaction path for oxygen intermediates to reduce adsorption energy and improve OER activity. The d-band center structure of the catalyst can be significantly affected by external forces such as ion etching and electric field strategies, and its catalytic performance can be further regulated. Zhu et al. used an electronic structure of NiMoO_4_ with a double cation etching strategy, and the absence of the double cation shifted the center of the Ni atom d-band up, resulting in better oxygen adsorption at the active site and further improving the OER activity of the NiMoO_4_ catalyst [121]. Li et al. developed a novel bifunctional electrocatalyst Ni/Co_3_O_4_ film by applying the strategy of electric field treatment [122]. After the electric field treatment, a conductive channel composed of oxygen vacancies is formed in the Co_3_O_4_ film, which significantly reduces the resistance of the system by nearly 2 × 10^4^ times. At the same time, the surface Ni metal electrode was partially oxidized to nickel oxide, which enhanced the catalytic activity. The Ni/Co_3_O_4_ material treated by the electric field showed excellent HER, OER and overall water cracking properties.

Two-dimensional materials have attracted increasing attention due to their unique physical, chemical and electronic properties, and the electrocatalytic performances of materials can be improved further with optimization of structure, conductivity, surface and interface. Zhao et al. prepared three-dimensional cobalt selenide electrodes with CoSe and Co_9_Se_8_ phases [123]. The charge state of Co and the electrocatalytic performance of the catalysts were controlled by controlling the mass ratio of Co to Se. Jia et al. prepared a layered amorphous MOF (Co-HAB) [124].This material exhibited excellent catalytic properties and stability when mixed with carbon black due to its open structure and dense active center. Lu et al. proposed a self-sustaining water splitting system with a two-dimensional Ti_3_C_2_T_*x*_ MXene perovskite oxide heterostructure; the material exhibits high electrocatalytic water splitting activity [125]. The performance of perovskite-based electrocatalysts was improved compared to previous reports. Abidi used density functional theory to study active sites of a two-dimensional structure MoS_2_ and found that these sites favored to the adsorption of OH^−^ ions, although the edge sites and substrate defects had a low thermodynamic overpotential (< 0.2 V) [126]. Wei et al. reported a simple plasma−rapid induced hydrothermal method to prepare MoS_2_ nanoparticle catalytic materials, which realized the adjustment of the nanosize of MoS_2_ materials [127]. Jiang et al. prepared CoS_2_ nanosheets with different grain sizes, and the best HER activity of the catalytic material was found in CoS_2_ nanosheets with porous structure by electrochemical performance test [128].

**Fig. 5:**
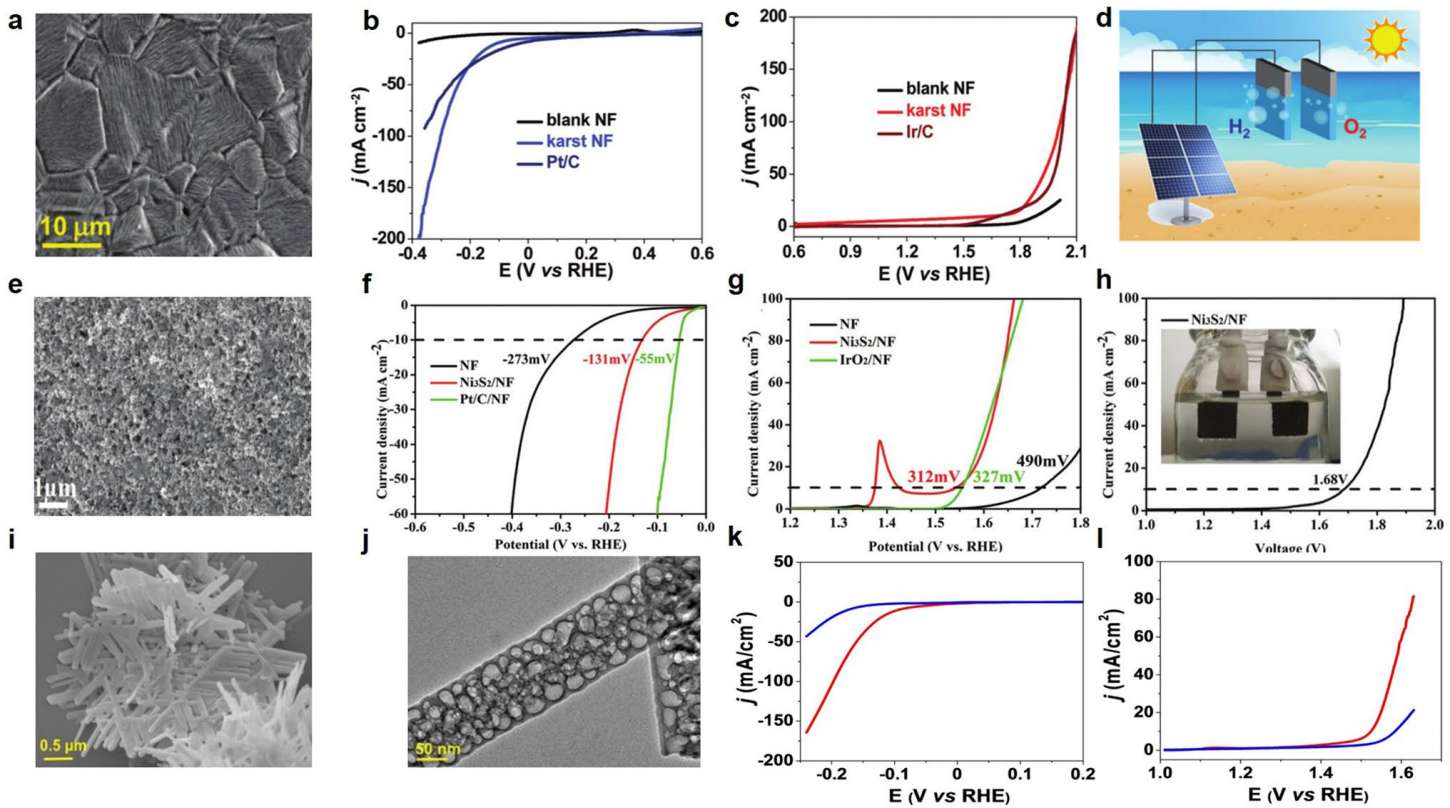
**a** SEM image of karst NF. **b** LSV curves of HER. **c** LSV curves of OER. **d** Illustration of the solar seawater splitting. Reproduced with permission [130]. Copyright 2020, Royal Soc Chemistry. **e** SEM image of Ni_3_S_2_/Ni foam. **f** LSV curves of the HER. **g** LSV curves of the OER. **h** LSV curve of water electrolysis in two electrodes. Reproduced with permission [131]. Copyright 2018, Royal Society of Chemistry. **i** SEM, and **j** TEM images of CoP. **k** LSV curves of CoP−acid (red) and CoP (blue) for HER, and **l** OER. Reproduced with permission [132]. Copyright 2020, WILEY−V C H VERLAG GMBH

Nickel-based foam catalyst is a frequently used catalyst for hydrogen production due to its high porosity and large specific surface area [129]. Researchers have fabricated various materials on foam nickel, such as alloy film and doped materials. The results show that these materials can effectively regulate electronic structure of catalysts and improve electrocatalytic water splitting process. Thus, the activity and stability of catalysts are improved, and it is suitable for industrial production with high currents. Our research group investigated a monolithic electrode based on nickel foam [130]. The electrode interior is nickel-metal, has a tower surface Ni/α-Ni (OH)_2_ heterostructure with karst characteristics (Fig. 5a), and exhibits high hydrogen evolution and oxygen evolution reactivity in neutral media (Fig. 5b, c). In combination with ordinary photovoltaic cells, water electrolysis cells with bifunctional electrodes can achieve cracking of natural seawater (Fig. 5d). Inexpensive, flexible, robust and readily available, this dual-function electrode is ideal for water cracking applications in the hydrogen economy. Liu et al. prepared nickel sulfide (Ni_3_S_2_) thin film (Ni_3_S_2_/NF) by dropping a sulfur ethanol solution on nickel foam and annealing at high temperatures [131]. The material provides a large number of active sites due to the large specific surface area of its structure (Fig. 5e). Both HER and OER showed good performance and stability in alkaline media (Fig. 5f−h). Zhang et al. synthesized CoP from Co(OH)F precursor through continuous phosphorylation and acid etching [132]. The 3D structure of the obtained CoP consists of zero-dimensional porous CoP rods, which are woven together to form 2D grid plates and then stacked together to form 3D structures (Fig. 5i, j), and both HER and OER have good catalytic properties (Fig. 5k, l).

**Fig. 6:**
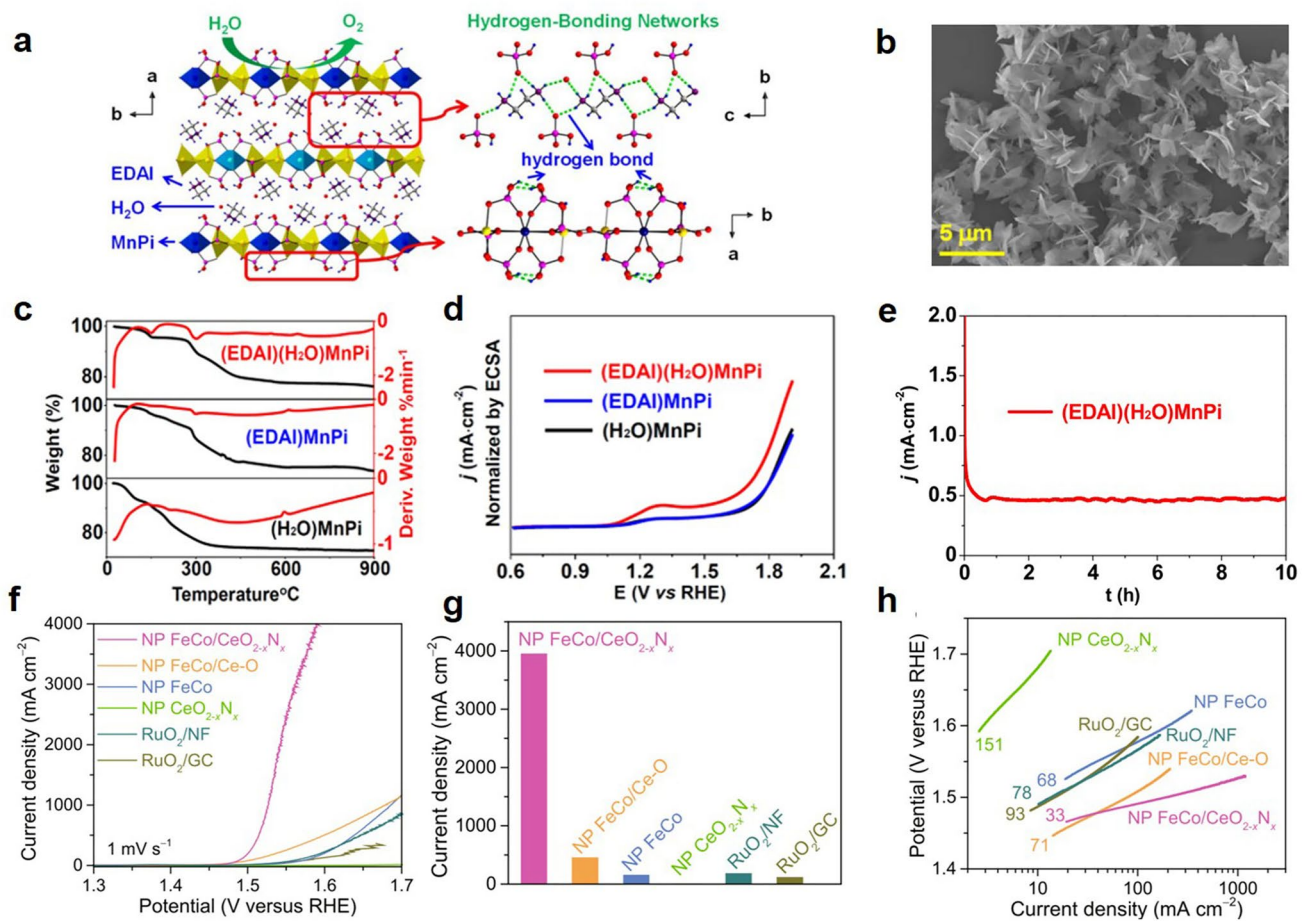
**a** Structure diagram. **b** SEM image of (EDAI) (H_2_O)MnPi. **c** Thermogravimetric analysis. **d** LSV curves. **e** Constant voltage electrolysis. Reproduced with permission [137]. Copyright 2020, Chinese Electronic Periodical. **f** LSV curves of the HER. **g** Comparison of current densities at overpotential of 360 mV. **h** Tafel plots. Reproduced with permission [42]. Copyright 2023, NATURE PORTFOLIO

The rich structural interfaces of this material enable it to provide more hydrogen adsorption sites and exhibit high activity in alkaline media. Zhao et al. reported on NiO/Ni heterogeneous catalysts supported on carbon nanotubes (CNTs) [133]. Zhu et al. prepared Ni-based core–shell structure catalyst on foam nickel matrix, which can expose more interfacial active sites and have efficient charge transport [134–136]. The oxygen-releasing center in nature is a manganese-containing complex, and manganese-based catalysts have attracted extensive research. Our research group prepared (EDAI)(H_2_O) MnPi (Fig. 6b) with a rich and continuous hydrogen bonding network formed by ethylenediamine ions and water molecules between layers (Fig. 6a) [137]. The hydrogen bonding network in this material accelerated the proton transfer rate and promoted electrocatalytic water oxidation with excellent catalytic activity (Fig. 6c−e). Zeng et al. established a multistage nanoporous alloy/nitrogen oxide-laminated composite electrode, which showed excellent electrocatalytic performance in alkaline solutions due to the provision of abundant electroactive sites and three-dimensional bicontinuous nanopores at the CoFeOOH/CeO_2−*x*_N_*x*_ interfaces (Fig. 6f−h) [42].

**Fig. 7:**
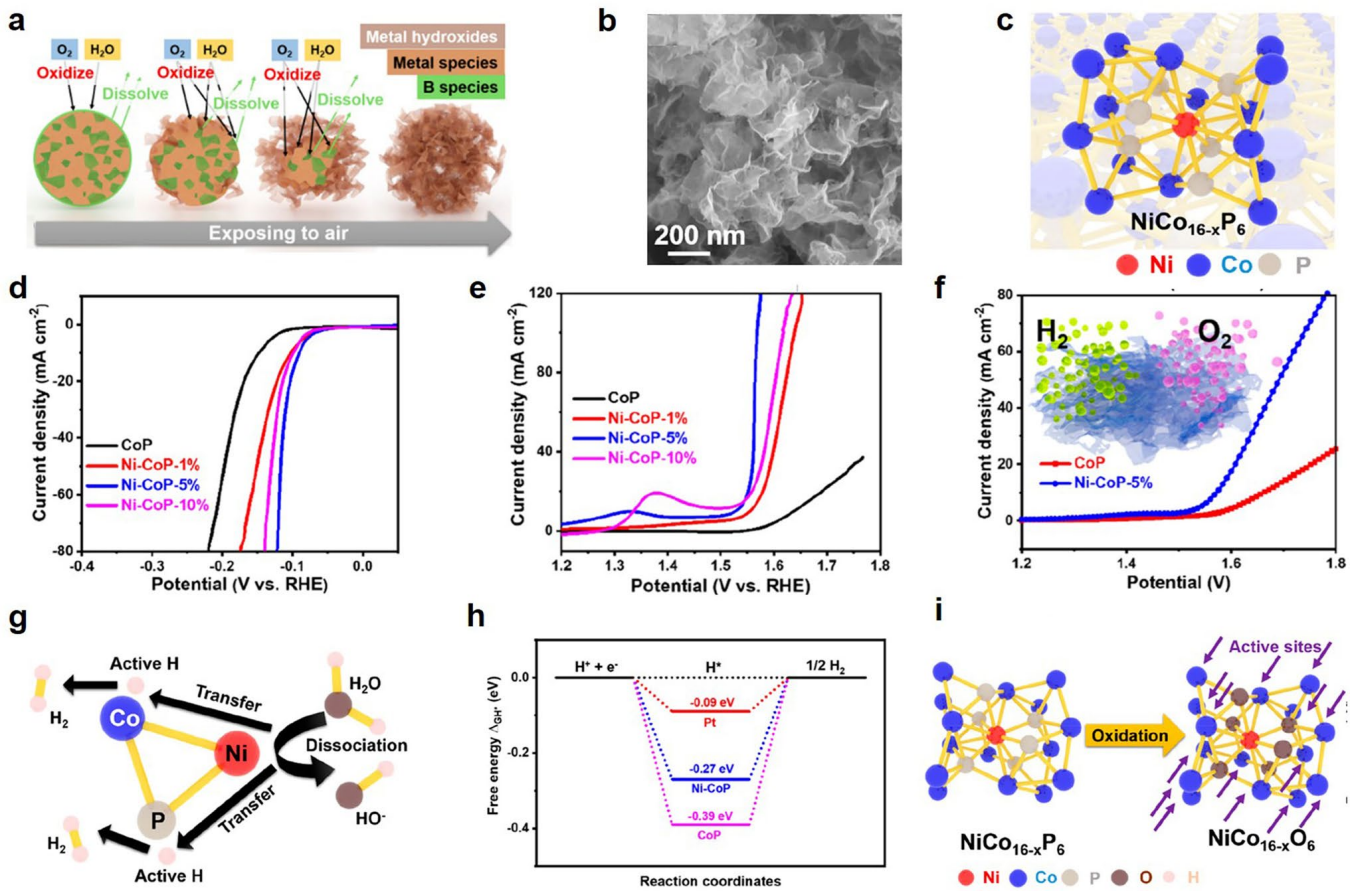
**a** Schematic illustration of formation mechanism of Ni−Co(OH)_2_ nanosheets. **b** SEM image of Ni−CoP−5%. **c** Schematic structure of NiCo_16−*x*_P_6_. **d** LSV curves of HER. **e** LSV curves of OER. **f** LSV curves of overall water separation. **g** HER mechanism study of NiCo_16−*x*_P_6_. **h** Free energy diagram of Ni−CoP. **i** Oxidized structure of NiCo_16−*x*_P_6_. Reproduced with permission [138]. Copyright 2021, American Chemical Society

Element doping can significantly regulate the catalytic activity of the catalyst. Zhao et al. prepared ultrathin nickel-doped CoP porous nanosheets (Fig. 7a−c) using a boron-assisted " release and oxidation " reaction pathway to form Ni−Co(OH)_2_ nanosheets [138]. This Ni−CoP catalytic electrode exhibited excellent HER and OER performance (Fig. 7d, e). Introduction of nickel into CoP to form atomic impurity metal sites in the center of NiCo_16−x_P_6_ can significantly improve the overall electrochemical decomposition performance of water. Therefore, we propose a method to synergistically improve HER from atomic impurity metal sites centered on NiCo_16−*x*_P_6_ (Fig. 7g). The free energies of CoP and NiCoP are shown in Fig. 7h, confirming the enhanced adsorption of H on the Ni−CoP surface during the HER process. However, the catalyst center undergoes an oxidation reaction to form oxidized NiCo_16−*x*_O_6_ sites before participating in the OER reaction process (Fig. 7i). The addition of oxygen and nickel atoms reduces the oxygenophilicity of Co atoms and decreases ability to bind oxygen intermediates, making it a flawless active site for OER. Wang et al. prepared FeCoP_2_ co-doped hollow carbon composites, which possessed excellent dual-activity water electrolysis catalytic activity [139].

#### Metal-Free Catalyst

Heteroatom-doped carbon materials are now widely studied metal-free catalysts. The performance of the catalyst is optimized by doping other nonmetallic heteroatoms to improve the constitutive site activity of the catalytic material [140–142]. Typically, metal-free elements such as N, B and S are incorporated into them [143, 144]. Yang et al. used electrochemical intercalation of commercial polyacrylonitrile-based carbon fibers, successfully embedding sulfur atoms into the carbon lattice, resulting in a surface morphology similar to graphene [145]. Zhang et al. prepared N-, P- and F-doped graphene catalysts using ammonium hexafluorophosphate as a nitrogen, phosphorus, and fluorine source through thermal decomposition [146]. The results showed that the catalyst exhibited excellent catalytic performance in both HER and OER.

### Economic Analysis of Catalysts for Hydrogen Production

Noble metals, noble metal alloys and their oxides are still the best performing catalysts. However, noble metal catalysts are more expensive to use, so it is important to develop high-performance and low-cost catalysts. Transition metal catalysts and nonmetallic catalysts have the advantages of low preparation cost, improving the catalytic activity of existing materials through design strategies such as size and morphology modulation, conductive carrier composite, atomic doping, crystalline phase modulation, amorphous engineering and interfacial engineering. The electrocatalytic properties of transition metal catalysts and nonmetal catalysts can be comparable to those of noble metal catalysts, which will be widely used.

### Comparison of Three Types of Catalysts

**Fig. 8:**
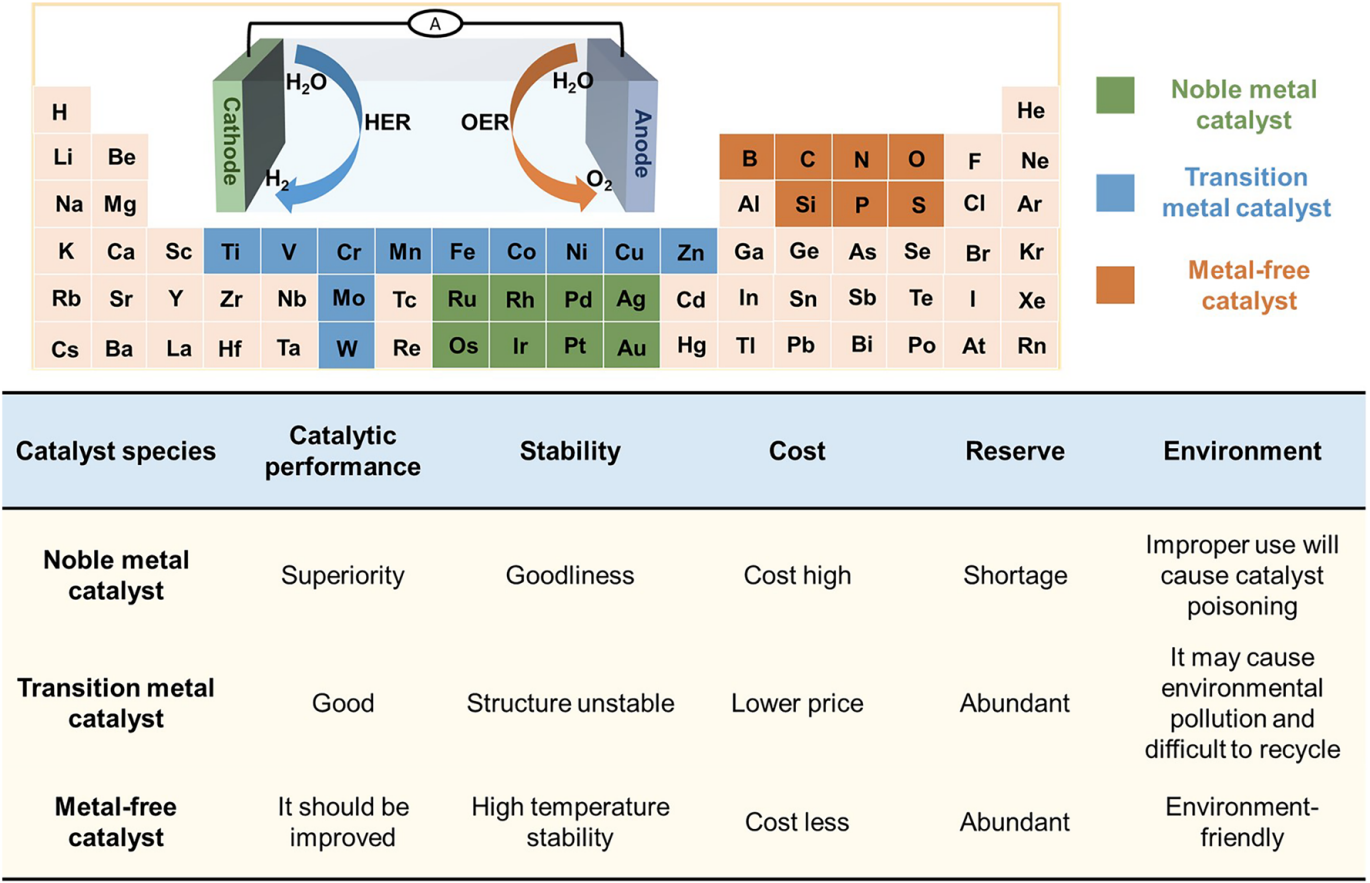
Comparison of different types of catalysts

The development of efficient and low-cost catalysts is a crucial step for hydrogen production by electrolysis of water. Noble metal catalysts are difficult to support large-scale applications due to their high cost and low storage. Transition metals and metal-free have low costs and sufficient storage capacity, making them ideal materials to replace noble metal catalysts. Figure 8 compares different types of catalysts. However, transition metal catalysts have unstable structures and complex catalytic mechanisms, and HER activity of metal-free catalysts should be improved compared with noble metal catalyst. The HER catalyst needs to be further studied, and the catalyst control strategy needs to be further refined. In addition to electrochemical parameters such as overpotential and Tafel slope, their evaluation parameters should also be further standardized. Specific preparation methods and actual environments should also be considered.

## Electrolyte for Water Electrolysis

In the electrolysis of water for hydrogen production, due to the fact that water is a weak electrolyte and the actual current is small, other electrolytes are generally added. The electrolyte acts as a charge carrier for charge transfer, and the electrical energy causes water molecules to break the balance and split into hydrogen and oxygen. The total reaction is H_2_O → H_2_ + O_2_, and the semi-reaction varies depending on the electrolyte [147–151]. The selection of electrolytes will affect the lifespan, energy consumption and cost of hydrogen production equipment for electrolysis of water. In the development process of electrolytes, it is necessary to comprehensively study the compatibility between electrolytes and catalysts, as well as the compatibility between electrolytes and energy fluctuations. The demand for hydrogen energy will continue to grow in future, so the electrolyte in water electrolysis for hydrogen production is attracting a lot of attention. Researchers are conducting in-depth studies of electrolytes from a variety of perspectives.

Depending on the electrolyte, it can be classified as using an alkaline solution [152], proton exchange membrane [153, 154], solid oxide [155–157], small molecule solution [158, 159], seawater [160–163], and so on. Alkaline solution electrolyte has low cost, high corrosiveness, short equipment life, and is the most mature technology. Proton membrane electrolytes have high efficiency and high cost and are a relatively mature technology. Solid oxide electrolyte has poor durability and slow start-up speed and is still at an experimental stage. The technology of using small molecule solutions and seawater as electrolytes has strong practicality, but it is still at the experimental research stage [164].

### Reaction Mechanism of Water Electrolysis

Hydrogen is obtained at the cathode and oxygen is gotten at the anode. Under different electrolytes, the chemical formula of the two half-reactions of the anode and cathode changes [165].

1. Acidic medium
  $${\text{Anode}}:{\text{2H}}_{{2}} {\text{O }}({\text{l}}) - {\text{4e}}^{ - } \to {\text{4H}}^{ + } ({\text{aq}}) + {\text{O}}_{{2}} \left( {\text{g}} \right)$$
  $${\text{Cathode}}:{\text{4H}}^{ + } \left( {{\text{aq}}} \right) + {\text{4e}}^{ - } \to {\text{2H}}_{{2}} \left( {\text{g}} \right)$$
2. Alkaline medium
  $${\text{Anode}}:{\text{4OH}}^{ - } ({\text{aq}}) - {\text{4e}}^{ - } \to {\text{2H}}_{{2}} {\text{O }}({\text{l}}) + {\text{O}}_{2} \left( {\text{g}} \right)$$
  $${\text{Cathode}}:{\text{2H}}_{{2}} {\text{O }}({\text{l}}) + {\text{4e}}^{ - } \to {\text{4OH}}^{ - } \left( {{\text{aq}}} \right) + {\text{2H}}_{2} \left( {\text{g}} \right)$$

Acidic conditions facilitate the migration of hydrogen ions and facilitate the synthesis and evolution of hydrogen, but the higher corrosiveness reduces the durability of the electrolysis equipment. Most inexpensive transition metal catalysts are susceptible to corrosion in strongly acidic environments. The efficiency of hydrogen production by electrolysis in alkaline electrolytes is lower than that in acidic electrolytes, and there is lighter corrosive effect on electrodes and electrolytic equipment [166].

#### Mechanism of HER

1. Volmer process
  $${\text{H}}^{ + } + {\text{e}}^{ - } \to {\text{H}}^{*}\left( {{\text{Acidic}}\;{\text{medium}}} \right)$$
  $${\text{H}}_{{2}} {\text{O}} + {\text{e}}^{ - } \to {\text{H}}^{*} + {\text{OH}}^{ - } \left( {{\text{Neutral}}\;{\text{or}}\;{\text{alkaline}}\;{\text{electrolyte}}} \right)$$
2. Heyrovsky process
  $${\text{H}}^{*} + {\text{H}}^{ + } + {\text{e}}^{ - } \to {\text{H}}_{{2}} \left( {{\text{Acidic}}\;{\text{medium}}} \right)$$
  $${\text{H}}^{*} + {\text{H}}_{{2}} {\text{O}} + {\text{e}}^{ - } \to {\text{H}}_{{2}} + {\text{OH}}^{ - } \left( {{\text{Neutral}}\;{\text{or}}\;{\text{alkaline}}\;{\text{electrolyte}}} \right)$$
3. Tafel process
  $${\text{H}}^{*} + {\text{H}}^{*} \to {\text{H}}_{{2}} \left( {{\text{Full}}\;{\text{pH}}\;{\text{range}}} \right)$$

In the acidic HER process, H* is firstly adsorbed through the electrochemical reduction process (Volmer process), followed by H* binding protons and electrons (Heyrovsky process) or direct binding of two molecules of H* (Tafel process) and desorption to form H_2_. However, in alkaline media, H^+^ is first formed by the dissociation of water, the Volmer process, and then H* desorption to form H_2_, due to the lack of H^+^. In general, the Gibbs free energy of H*, Δ*G*_H*_, is considered to be an important parameter to describe HER performance. If Δ*G*_H*_ is negative, it means that the catalyst surface is more conducive to H* binding and Volmer reaction is easy to proceed. When Δ*G*_H*_ is more negative, the adsorption of H* is more firm, which is not conducive to the Heyrovsky or Tafel steps. When the Δ*G*_H*_ of the catalyst is large and correct, it indicates that the adsorption of H* on the catalyst surface is weaker and the Volmer reaction is difficult to occur, which leads to slower overall reaction kinetics. Therefore, a highly active HER catalyst should have a better Δ*G*_H*_, that is, the closer Δ*G*_H*_ is to zero, the more beneficial the HER process. However, in alkaline media, in addition to the adsorption and desorption of H*, water adsorption and activation also determine the HER performance of the catalyst to a certain extent. The lower water adsorption energy represents the good affinity of water molecules on the catalyst surface, which is conducive to the subsequent reaction. A small water activation energy indicates a fast hydrolysis rate. Therefore, in alkaline media, the ideal HER catalyst needs to have moderate water adsorption energy and water activation energy in addition to having a Δ*G*_H*_ close to zero.

#### Mechanism of OER

1. Acidic or neutral medium
  $${\text{H}}_{{2}} {\text{O}} + ^{*} \to ^{*}{\text{OH}} + {\text{H}}^{ + } + {\text{e}}^{ - }$$
  $$^{*}{\text{OH}} \to ^{*}{\text{O}} + {\text{H}}^{ + } + {\text{e}}^{ - }$$
  $$^{*}{\text{O}} + {\text{H}}_{{2}} {\text{O}} \to ^{*}{\text{OOH}} + {\text{H}}^{ + } + {\text{e}}^{ - }$$
  $$^{*}{\text{OOH}} \to {\text{O}}_{{2}} + {\text{H}}^{ + } + {\text{e}}^{ - }$$
2. Alkaline medium
  $${\text{OH}}^{ - } + ^{*} \to ^{*}{\text{OH}} + {\text{e}}^{ - }$$
  $$^{*}{\text{OH}} + {\text{OH}}^{ - } \to ^{*}{\text{O}} + {\text{H}}_{{2}} {\text{O}} + {\text{e}}^{ - }$$
  $$^{*}{\text{O}} + {\text{OH}}^{ - } \to ^{*}{\text{OOH}} + {\text{e}}^{ - }$$
  $$^{*}{\text{OOH}} + {\text{OH}}^{ - } \to {\text{O}}_{{2}} + {\text{H}}_{{2}} {\text{O}} + {\text{e}}^{ - }$$

The OER process, whether acidic or basic or neutral, goes through four basic steps to form *OH, *O and *OOH reaction intermediates in turn. Among the four basic steps, the biggest energy barrier is the decisive speed step of the OER reaction process, which also determines the reaction performance of the catalyst. According to Sabatier's principle, the binding ability of the reaction intermediate to the catalytic active site determines the OER activity of the catalyst. Therefore, either too strong or too weak binding capacity is detrimental to the OER kinetic process.

### Introduction to Common Electrolyzers

#### Alkaline Water Electrolysis

Alkaline water electrolysis (ALK/AWE) is used for large-scale hydrogen production. It is cheaper and has a conversion efficiency of 60%−80% and a cell operating voltage of 1.8−2.4 V [167]. The main equipment consists of a power supply, cathode and anode, diaphragm, electrolyte and electrolyzer box, and the electrolyte is usually a sodium hydroxide solution. The advantage of alkaline electrolyzers is that they can operate at low temperatures and do require transition metal catalysts to activate and produce hydrogen [168]. On the other hand, corrosion of the electrolysis electrodes is considered the main challenge due to the presence of alkaline solutions.

#### Proton Exchange Membrane Electrolysis

Proton exchange membrane (PEM) water electrolyzer is more efficient than alkaline electrolyzer and mainly use ion exchange technology. The electrolyzer consists mainly of a polymer film, a cathode and an anode. Due to the high proton conductivity, the PEM water electrolyzer can operate at much higher currents, thus increasing the electrolysis efficiency [169, 170]. With the advancement of proton exchange membrane and noble metal electrode technology, the cost of hydrogen production in polymer film electrolytes will be greatly reduced. Although PEM electrolyzer have been commercialized, they have some drawbacks, mainly the high investment cost and the high cost of both the membrane and noble metal-based electrodes [171, 172]. In addition, the lifetime of the PEM electrolyte is shorter than that of the alkaline one [173]. In the future, the hydrogen production capacity of PEM electrolyzer will need to be significantly increased.

#### High-Temperature Solid Oxide Electrolysis

The solid oxide electrolysis (SOEC) needs work at high temperatures, and part of the electrical energy can be replaced by heat, with high efficiency and low cost [174]. The efficiency of the solid oxide electrolyzer is the highest among the three types of electrolyzer, and the waste heat after the reaction can be recovered by the turbine and cooling system to improve the efficiency, which can reach 90%. The main obstacle to the current industrial application of solid oxide electrolyzer is the long-term stability of the electrolyzer, and there are also problems of electrode aging and deactivation [175, 176].

#### Anion Exchange Membrane Electrolysis

Anion exchange membrane (AEM) electrolyzer is one of the more cutting-edge water electrolysis technologies. The principle is that the raw water enters from the cathode side of the AEM equipment. Water molecules participate in the reduction reaction at the cathode to obtain electrons and produce hydrogen and oxygen ions. Hydrogen and oxygen ions reach the anode through the polymer anion exchange membrane and participate in the oxidation reaction to lose electrons and produce water and oxygen. A certain amount of potassium hydroxide or sodium bicarbonate solution is sometimes added to the raw water as an auxiliary electrolyte, which helps to improve the working efficiency of the AEM electrolysis equipment [177–179].

The anion exchange membrane electrolysis of water for hydrogen production combines the advantages of alkaline water electrolysis and PEM electrolysis. It has higher current density and response speed and higher energy conversion efficiency. Moreover, the electrolyte used is pure water or low-concentration alkaline solution, which alleviates the corrosion of strong alkaline solution on the equipment [10]. In addition, AEM technology can also be used as a catalyst for Fe, Ni and other non-noble metal electrodes, and its device manufacturing cost is significantly reduced compared with PEM technology [180]. Compared with PEM electrolytic water technology, the device cost is significantly reduced. In general, this technology is superior to alkaline water electrolysis for hydrogen production, but it is still in the experimental research and development stage. Therefore, different water electrolysis technologies face different challenges such as cell performance, durability, membrane materials, catalysts and battery cost.

### Different Electrolytes for Water Electrolysis

#### Alkaline Solution as Electrolyte

Nicholison and Carlisle used strong alkaline solutions of KOH and NaOH as electrolytes for the production of hydrogen for the first time in 1800. This process was industrialized in the mid-twentieth century. Although its cost is relatively low, many studies have found that processes using alkaline solutions as electrolytes consume large amounts of freshwater resources and place demands on the high performance of OER catalysts in alkaline environments, resulting in huge energy consumption. The corresponding performance of various electrocatalysts in different solutions, such as acidic, neutral and alkaline solutions, is summarized in Table 1. At present, the research on alkaline solution as electrolyte technology at home and abroad focuses on the search for corrosion-resistant membrane electrode materials and suitable catalysts [181, 182]. David Aili et al. studied advanced alkaline electrolysis using ionic solvation polymer membrane as electrolytes, which greatly improved the stability of the polymer in alkaline environments [183]. Gao et al. proposed a self-sustaining control model of electrolysis units, aiming to maintain a stable internal working environment temperature and improve the electrolysis efficiency [184]. Wei et al. prepared an electrolyte of nano-carbon black/sodium hydroxide solution [185]. This research has shown that adding carbon black to the electrolyte increases hydrogen production by 23.62%. Lai et al. synthesized NiO nanoparticles, multi-walled carbon nanotubes (MWCNTs) and low-layer molybdenum sulfide nanosheets, which showed excellent catalytic performance [186]. Patolsky et al. and their group [187] proposed a single-step solid-state method for the conversion of a nickel-based substrate to a single crystalline nickel sulfide nanoplate array. The effect of the transition temperature on the crystal growth direction is also found, so that the chemical state of the catalyst surface can be controlled. The electrocatalytically active Ni^3+^ concentration on the surface of nickel-based sulfide formed at 450 °C is enhanced and the electron density around the sulfur atom is reduced, which is most suitable for efficient hydrogen production. Nickel-based sulfide electrocatalyst showed excellent electrocatalytic properties of oxygen and hydrogen evolution.

**Table 1:** Electrochemical properties of electrocatalysts in acidic, neutral and alkaline solutions

Type of catalyst	Catalysts	Electrolyte	Overpotential HER (mV)	Tafel slope (mV dec^−1^)	Stability	References
Transition metal	Ni_2_P–Ni_12_P_5_@ Ni_3_S_2_	1 M KOH	*η*_10_ = 32	85	24 h	[188]
		0.5 M H_2_SO_4_	*η*_10_ = 46	78	24 h	
		1.0 M PBS	*η*_10_ = 34	146	24 h	
	CoFe–P NAs/IF	1 M KOH	*η*_10_ = 40	62.0	200 h	[189]
	Cr, Fe–CoP/NF	1 M KOH	*η*_10_ = 27	58	400 h	[190]
	NiP/H_*x*_WO_3_	0.5 M H_2_SO_4_	*η*_500_ = 280	72.5	110 h	[191]
	MoNi_4_**-**NiO	1 M KOH	*η*_10_ = 41	86.7	100 h	[192]
	FeP/Ni_2_P	1 M KOH	*η*_10_ = 46	50.5	50 h	[193]
	Co–Fe–P/CeO_2_ HHRs	1 M KOH	*η*_10_ = 69.7	90.1	30 h	[194]
	fcc Fe–Pd	1 M KOH	*η*_10_ = 58	88	48 h	[195]
	Mo_2_C@CoO/N-CNFs	1 M KOH	*η*_10_ = 115	76	30 h	[117]
	Mn–NiCoP	1 M KOH	*η*_100_ = 148	53	240 h	[119]
	Co_2_P/CoN-in-NCNTs	0.5 M H_2_SO_4_	*η*_10_ = 98	57	96 h	[116]
	NiS_*x*_-24 h	1.0 M PBS	*η*_10_ = 173	209.5	1000 cycles	[118]
	Ni/MoO_2_@CN	1 M KOH	*η*_10_ = 33	45	200 h	[196]
	NIS-450	1 M KOH	*η*_10_ ≈ 98	–	10 h	[187]
	Ni_*x*_S_*y*_@MnO_*x*_H_*y*_/NF	1 M KOH	*η*_100_ = 270	95.1	100 h	[110]
	FeNiZn/FeNi_3_@NiFe	1 M KOH	*η*_100_ = 245	47.3	400 h	[112]
	Cu_50_W_50_	1 M KOH	*η*_10_ = 65	–	200 h	[197]
Precious metal	Ru-WO_3−*x*_	1.0 M PBS	*η*_10_ = 19	78	30 h	[198]
	La_2_Sr_2_PtO_7+δ_	0.5 M H_2_SO_4_	*η*_10_ = 13	19	50 h	[199]
	Os–OsSe_2_	0.5 M H_2_SO_4_	*η*_10_ = 26	31	10 h	[200]
		1 M KOH	*η*_10_ = 23	–	–	
	Ru–P(Ir 3 at.%)	0.5 M H_2_SO_4_	*η*_10_ = 33	33	24 h	[201]
		1 M KOH	*η*_10_ = 7	–	–	
	Ru_3_Sn_7_	0.5 M H_2_SO_4_	*η*_10_ = 28	22	50 h	[202]
		1 M KOH	*η*_10_ = 27	40	–	
	0.04W-Mo_2_C-725-Ar	1 M KOH	*η*_10_ = 99.86	66	24 h	
	PtFeCoNiCu HEA	0.5 M H_2_SO_4_	*η*_10_ = 30.7	28.1	–	[203]
	PdH_*x*_@Ru	1 M KOH	*η*_10_ = 30	30	25 h	[204]
	Pt/A-NiCo LDH/NF	1 M KOH	*η*_10_ = 16	38.8	40 h	[205]
	RhNi NPNWs	1 M KOH	*η*_10_ = 43.1	20.3	25 h	[206]
	RuP@RuP_2_/C	1 M KOH	*η*_10_ = 11.6	27.5	20 h	[207]
	Ir@NBD-C	1 M KOH	*η*_10_ = 7	–	5000 cycles	[84]
		0.5 M H_2_SO_4_	*η*_10_ = 8	–	5000 cycles	
		1.0 M PBS	*η*_10_ = 37	–	5000 cycles	
	Ru@Ni-MOF/NF	1 M KOH	*η*_10_ = 22	42	24 h	[208]
	u-Ru-1/C	1 M KOH	*η*_10_ = 31	26	10 h	[209]
	RuRh_2_ bimetallene	1 M KOH	*η*_10_ = 24	34	–	[210]
	FeIr/NF	1 M KOH	*η*_10_ = 6.2	40.94	384 h	[211]
	hcp Ir−Ni	1 M KOH	*η*_10_ = 17	16	20 h	[212]
	Ru–O–Mn/CPD	1 M KOH	*η*_10_ = 35	20.7	40 h	[213]
	NiO/RuO_2_	1 M KOH	*η*_1000_ = 178	42.0	72 h	[214]
	Ru_0.5_Ir_0.5_	1 M KOH	*η*_10_ = 28	14	100 h	[215]
		1.0 M PBS	*η*_10_ = 16	24	100 h	
		0.5 M H_2_SO_4_	*η*_10_ = 4	13	400 h	
	Ru/Co@NC	1 M KOH	*η*_10_ = 10	23	30 h	[216]
		1.0 M PBS	*η*_10_ = 283	143	30 h	
		0.5 M H_2_SO_4_	*η*_10_ = 50	46	30 h	
Metal-free	RH-CG	0.5 M H_2_SO_4_	*η*_10_ = 9	31	10 h	[217]
	N-VG	0.5 M H_2_SO_4_	*η*_10_ = 290	121	10 h	[218]
	TAGDY	0.5 M H_2_SO_4_	*η*_10_ = 82	74.13	10 h	[219]
	(rGO)/SiO_2_	0.5 M H_2_SO_4_	*η*_10_ = 134	103	1000 cycles	[220]
	Ultrathin GDY/CF	0.5 M H_2_SO_4_	*η*_10_ = 68	41.6	1000 cycles	[221]
	BCN@GCs	0.5 M H_2_SO_4_	*η*_10_ = 333	39.0	24 h	[222]
	Functionalized CNT	0.5 M H_2_SO_4_	*η*_10_ = 135	38.0	700 cycles	[223]
	10% F/BCN	0.5 M H_2_SO_4_	*η*_10_ = 220	87.0	12 h	[224]
	N, P, O-porous carbon	1 M KOH	*η*_10_ = 179	93	5000 cycles	[225]
	Conjugated poly−indigo	1 M KOH	*η*_10_ = 270	56	24 h	[226]
	CDs/CNHs	0.5 M H_2_SO_4_	*η*_10_ = 290	97	10,000 s	[216]
	P-rGO-g-C_3_N_4_	0.5 M H_2_SO_4_	*η*_10_ = 146	122.5	500 cycles	[227]
	N, P-carbon	1 M HClO_4_	*η*_10_ = 260	175	10,000 cycles	[228]
	N-doped carbon	1 M KOH	*η*_10_ = 198.6	95.2	2000 cycles	[229]

**Fig. 9:**
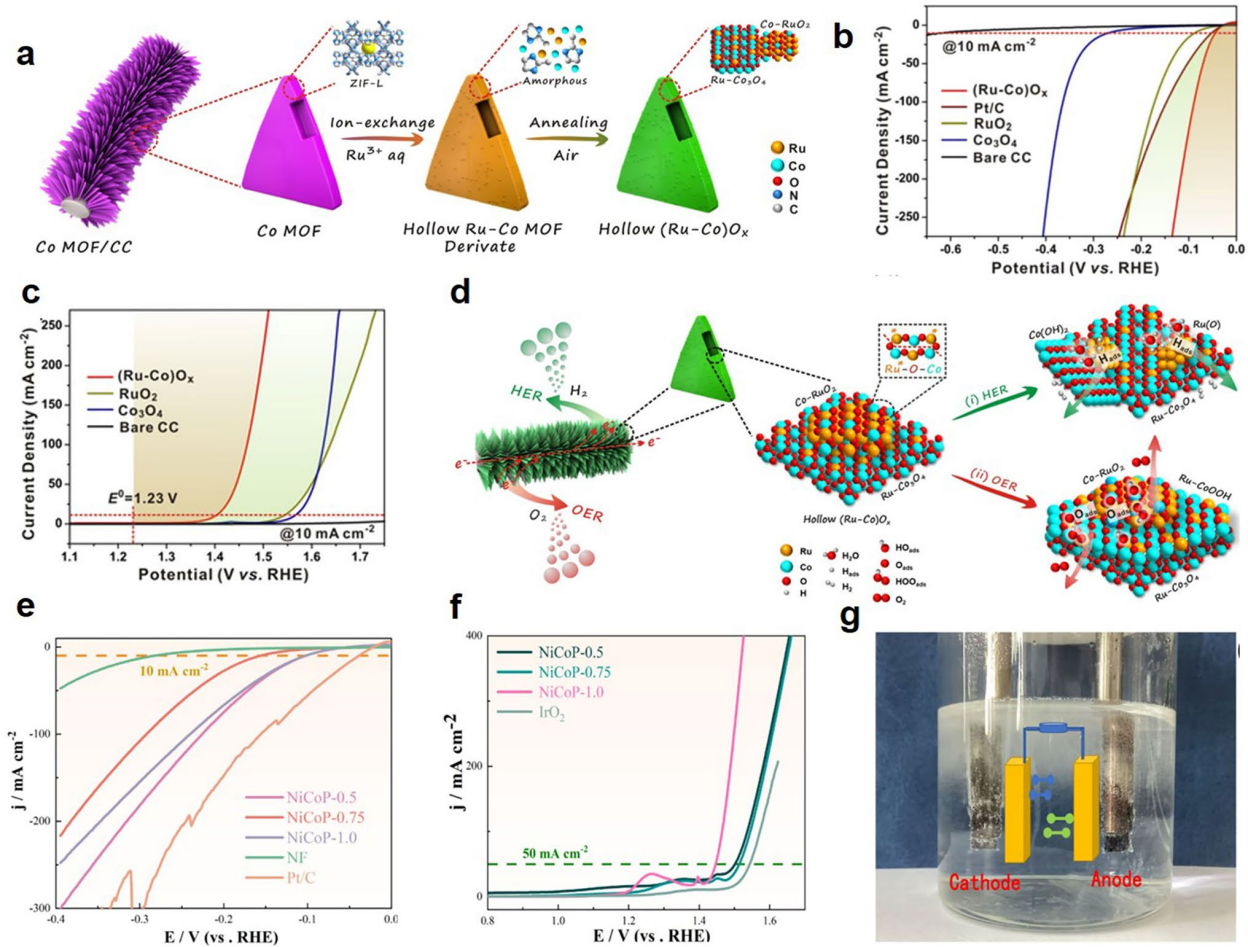
**a** Schematic diagram of the sample preparation process. **b** LSV curves of HER. **c** LSV curves of OER. **d** Schematic diagram of enhanced water decomposition mechanism. Reproduced with permission [231]. Copyright 2020, WILEY−V C H VERLAG GMBH. **e** LSV curves of HER. **f** LSV curves of OER. **g** Schematic diagram of double electrode water electrolysis. Reproduced with permission [232]. Copyright 2023, Elsevier BV

Nady et al. investigated the effect of doping molybdenum, chromium and iron metal elements in alkaline electrolytes on the electrocatalytic activity of nickel-based alloys [230]. Wang et al. prepared oxide–cobalt oxide (Ru–Co)O_*x*_ (Fig. 9a) [231]. This co-doping shows a good electronic structure and has the advantage of water electrolysis in the microstructure, and the (Ru–Co)O_*x*_ nanoarrays have excellent electrochemical properties when used in alkaline environments as catalytic materials for both hydrogen and oxygen precipitation (Fig. 9b–d). Wang et al. synthesized NiCoP nanosheets on nickel foam [232]; the NiCoP-1.0 catalyst was confirmed to have good electrochemical performance and structural stability in alkaline electrolytes (Fig. 9e–g).

#### Seawater as Electrolyte

At present, electrolyzed water mainly uses freshwater as raw material, which undoubtedly exacerbates the shortage of freshwater resources on the earth [233–237]. By regulating the composition of electrolyte, the hydrogen production efficiency can be continuously improved and the energy consumption can be gradually reduced. The use of abundant seawater instead of freshwater to produce the electrolyte is expected to solve the problem of freshwater consumption. Currently, many catalysts have been studied in seawater electrolysis so far, and lots of work about small organic molecules oxidation have also been reported in seawater [238–240]. However, direct decomposition of untreated seawater is still difficult due to the neutral, unbuffered nature of the electrolytic medium and the presence of high chloride concentrations. New scientific and technological developments are urgently needed to direct cleavage of seawater for sustainable hydrogen production [241]. The corresponding properties of various electrocatalysts in seawater are summarized in Table 2.

**Table 2:** Electrochemical properties of electrocatalysts in seawater solution

Type of catalyst	Catalysts	Electrolyte	Overpotential OER (mV)	Overpotential HER (mV)	Overall (V)	Stability	References
Transition metal	NiFe LDH/NF	Seawater	*η*_100_ = 247	–	*E*_10_ = 1.477	96 h	[242]
	(Ni, Fe, Mo)OOH/NF	1 M KOH + seawater	*η*_400_ = 416	–	–	80 h	[243]
	NiFe−CuCo LDH	6 M KOH + seawater	*η*_500_ = 283	–	–	500 h	[244]
	3D Mn-doped Ni_2_P/Fe_2_P	1 M KOH + 0.5 M NaCl	*η*_500_ = 325	*η*_500_ = 425	*E*_500_ = 2.02	120 h	[245]
	NiFe-LDH-S	1 M KOH + 0.5 M NaCl	*η*_100_ = 296	–	–	12 h	[246]
	Ni_3_S_2_/Co_3_S_4_	1 M KOH + seawater	*η*_100_ = 280	–	*E*_500_ = 1.94	> 100 h	[247]
	S-NiMoO_4_@NiFe-LDH	1 M KOH + 0.5 M NaCl	*η*_100_ = 315	*η*_100_ = 220	*E*_100_ = 1.68	20 h	[235]
	S-(Ni, Fe)OOH	Seawater	*η*_500_ = 392	–	*E*_500_ = 1.837	24 h	[248]
	S-NiFeO_*x*_H_*y*_/CC	1 M KOH + 0.5 M NaCl	*η*_100_ = 250	–	–	24 h	[249]
	NiFe–NiS_*x*_–NF	1 M KOH + 0.5 M NaCl	*η*_400_ = 300	–	*E*_400_ = 2.1	> 1000 h	[250]
	GO@Fe@Ni-Co@NF	1 M KOH + seawater	*η*_500_ = 303	–	*E*_500_ = 1.94	378 h	[251]
	Ni_3_S_2_–MoS_2_–Ni_3_S_2_/NF	1 M KOH + 0.5 M NaCl	*η*_100_ = 330	–	*E*_100_ = 1.80	> 100 h	[252]
	Fe–Ni_2_Pv	1 M KOH + seawater	*η*_1000_ = 180	*η*_3000_ = 286	*E*_1000_ = 1.68	3000 cycles	[253]
	Cr–Co_x_P	1 M KOH + seawater	*η*_20_ = 268	*η*_1000_ = 292	–	140 h	[254]
	F-FeCoPv@IF	1 M KOH + seawater	*η*_1000_ = 370	*η*_1000_ = 210	*E*_1000_ = 1.94	> 100 h	[255]
	MoNi/NiMoO_4_	1 M KOH + 0.5 M NaCl	–	*η*_10_ = 256	–	24 h	[256]
	NiTe–NiCoN	1 M KOH + seawater	*η*_10_ = 211	*η*_10_ = 68	*E*_400_ = 1.84	100 h	[257]
	NiFeS/NF	1 M KOH + seawater	*η*_500_ = 300	*η*_500_ = 347	*E*_100_ = 1.85	50 h	[258]
	NiFeCoLDH	1 M KOH + seawater	*η*_100_ = 304	–	*E*_840_ = 1.7	80 h	[259]
	Fe-Ni_2_P	6 M KOH + seawater	*η*_100_ = 266	–	–	600 h	[260]
	CuB_x_@PU	1 M KOH + 0.5 M NaCl	*η*_10_ = 136	*η*_10_ = 70	*E*_100_ = 1.45	20 h	[261]
	NiS_2_pS_*x*_^surface^	Natural seawater	*η*_10_ = 197	–	*E*_10_ = 1.39	40 h	[262]
	Cr_2_O_3_–CoO_*x*_	Natural seawater	–	–	*E*_1000_ = 1.87	100 h	[263]
	Na_2_Co_1−*x*_Fe_*x*_P_2_O_7_/C@CC	1 M KOH + 0.5 M NaCl	*η*_100_ = 480	–	*E*_100_ = 1.6	100 h	[264]
	Mo_5_N_6_	Natural seawater	–	*η*_10_ = 257	–	100 h	[265]
	Co–Fe_2_P	1 M KOH + 0.5 M NaCl	*η*_50_ = 251	*η*_50_ = 117	*E*_100_ = 1.69	22 h	[266]
Precious metal	Ag/NiFe LDH	1 M KOH + seawater	*η*_1000_ = 303	–	–	1000 h	[267]
	Ru/Cd_0.02_Se_4_	1 M KOH + seawater	–	*η*_10_ = 6.3	–	50 h	[268]
	Ir_1_/Ni_1.6_Mn_1.4_O_4_	0.5 M KOH + seawater	*η*_100_ = 330	–	*E*_500_ = 1.5	60 h	[269]
	Ru_1+NPs_/N–C	1 M KOH + seawater	–	*η*_10_ = 58	–	24 h	[270]
	NiFe-LDH@Ag	1 M KOH + seawater	–	–	*E*_400_ = 1.96	> 5000 h	[267]
	Pt@CoMo_2_S_4_-NG/NF	1 M KOH + seawater	–	*η*_10_ = 27	*E*_10_ = 1.54	100 h	[271]
	0.5Ru0.1Cu-GN1000	Natural seawater	–	*η*_10_ = 389	–	–	[272]
	Pt/GaN/Si	0.5 M NaCl	–	*η*_10_ = 150	–	4 h	[273]
	Co_3−*x*_Pd_*x*_O_4_	1 M KOH + 1 M PBS	*η*_10_ = 370		*E*_100_ = 2.65	250 h	[274]
	MIL-(IrNiFe)@NF	1 M KOH + seawater + 0.5 M N_2_H_4_	*η*_500_ = 220	–	*E*_1000_ = 0.69	24 h	[275]
	Pt_0.06_Ru_0.24_Ti_0.7_O_*x*_	0.5 M NaCl + 5 mM NaClO	–	–	–	500 h	[276]
	Ru/FeTeO	1 M KOH + seawater	–	*η*_1000_ = 244	*E*_1000_ = 1.81	150 h	[277]
	NiIr-LDH	1 M KOH + 0.5 M NaCl	*η*_100_ = 286	–	–	650 h	[278]
	Pt_a_t–CoP MNSs/CFC	Natural seawater	–	*η*_10_ = 13	–	24 h	[279]
	Pt–Ni@NiMoN/NF	1 M KOH + seawater	–	*η*_10_ = 11	–	200 h	[280]
	Co_6_W_6_C-2–600	3 M KOH + 3 M NaCl	*η*_10_ = 290	*η*_10_ = 50	*E*_10_ = 1.56	50 h	[281]
Metal-free	PA-PPy/CP	1 M KOH + 0.5 M NaCl	*η*_10_ = 569	–	–	–	[282]

Tahri et al. provided an effective method for electrolysis of seawater for hydrogen production by controlling the chemical processes of marine hydropower catalytic reactions [283]. Li et al. developed an efficient triple electrolysis water system by cracking seawater to produce hydrogen, oxygen and crystalline sodium chloride in alkaline electrolytes with high chloride content [284].

**Fig. 10:**
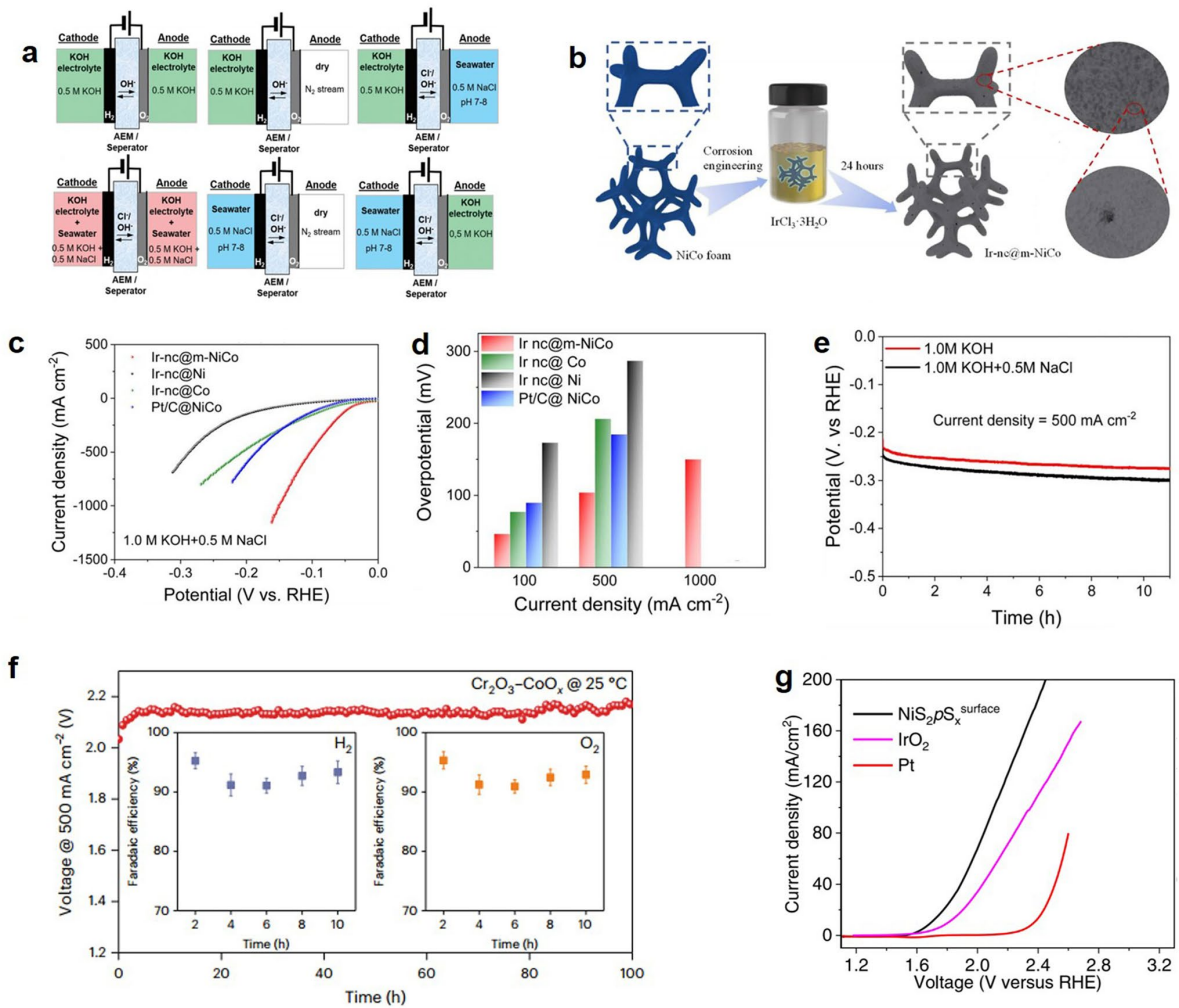
**a** Schematic diagram of different electrolytes used in AEM electrolytic cells. Reproduced with permission [285]. Copyright 2020, Royal Society of Chemistry. **b** Schematic illustration of the fabrication of Ir–nc@m–NiCo. **c** HER polarization curves. **d** Comparison of overpotential. **e** Long-term stability test. Reproduced with permission [286]. Copyright 2023, Royal Society of Chemistry.** f** Long-term stability test of Cr_2_O_3_–CoO_*x*_. Reproduced with permission [263]. Copyright 2023, Springer Nature. **g** OER polarization curves of NiS_2_pS_*x*_^surface^. Reproduced with permission [288]. Copyright 2023, Elsevier BV

At present, there are abundant seawater resources available for use in the environment. However, the cost of using this electrolyte is higher than other electrolytes, and impurities have a greater effect on the cracking reaction. Another challenge is how to effectively break down seawater without increasing its alkalinity. In order to bypass the limit electrode potential, Dresp et al. developed a new feeding method of electrolytic cell and compared it with traditional seawater and alkaline electrolytic cell electrolyte supply schemes (Fig. 10a) [285]. At present, this technology is still relatively immature, and more research is needed in this area. Yuan et al. developed Ir-NC@mNiCo (Fig. 10b) [286]. The catalyst exhibited significant HER performance in simulated alkaline seawater electrolytes (Fig. 10c, d). A slight increase in durability was observed after a 10-h test below 500 mA cm^−2^ (Fig. 10e). Zhou et al. by growing Ni–B catalysts in situ on the surface of hydrophilic filterable paper [287]. Overpotentials of only 32 and 300 mV were achieved during electrolytic water hydroxogenesis reactions (HER and OER), reaching current densities of 10 mA cm^−2^, with no degradation after > 3 days of stable operation at industrial current densities (> 500 mA cm^−2^). More fascinatingly, the catalyst is based on a filter paper that maintains its own filtration capacity, while also being self-filtering for seawater and sewage.

In recent years, a number of pioneering studies have reported direct splitting of seawater by different methods. Qiao et al. and their group [263] introduced a Lewis acid layer (Cr_2_O_3_) over a transition metal oxide catalyst to dynamically split water molecules and trap hydroxyl anions. This localized alkalinity generated in situ favors the kinetics of the reaction at both electrodes, avoiding chloride erosion and precipitate formation at the electrodes. Long-term stability of 100 h at 500 mA cm^−2^ was achieved by direct electrolysis of real seawater without alkalization or acidification (Fig. 10f). The results observed in the literature are quite encouraging. The results open up ambitious methods for directly splitting seawater. Patolsky et al. and their research group [262] prepared a single-crystal, high specific surface area, three-dimensional electrocatalyst based on chain sulfur–nickel polysulfides (NiS_2_pS_*x*_^surface^). By adjusting the flow rate and temperature of the catalyst, the electrocatalytic performance of the catalyst can be significantly improved. The overpotential of this catalyst was 460 mV, which was significantly lower than that of the IrO_2_ and Pt catalysts. It is important to note that such a low overpotential value was observed without the use of any base and buffer, representing the best value of neutral aqueous NaCl oxidation reported so far. When directly dissolving seawater without additives, a cell voltage of 1.39 V (10 mA cm^−2^) was observed. The industrial practical scale of 500 mA cm^−2^ is the lowest battery voltage reported so far. The electrocatalyst was also found to be an excellent OER catalyst under non-buffer neutral water conditions, exhibiting a significantly reduced overpotential (*η* = 320 mV) compared to currently accepted noble metal IrO_2_ catalysts (Fig. 10g). This overpotential value is below the evolutionary limit of chlorine under neutral conditions and provides a potential platform for electrocatalytic direct cleavage of seawater by intrinsic chloro-hydrophobic [288].

**Fig. 11:**
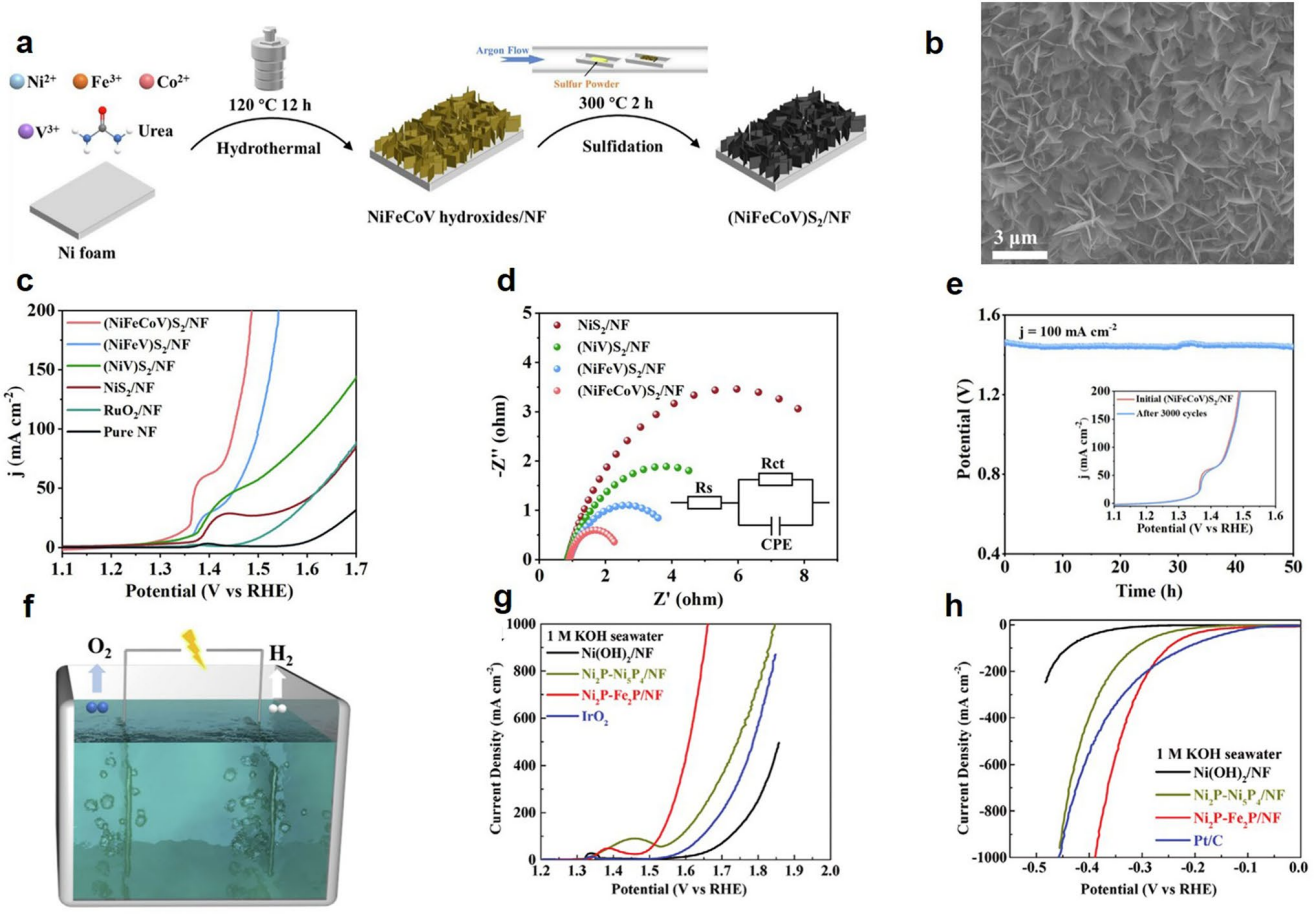
**a** Schematic diagram of sample synthesis. **b** SEM image of (NiFeCoV)S_2_. **c** LSV curves of OER. **d** Nyquist plots. **e** OER chronopotential curves for this sample at constant current density. **f** Schematic diagram of double electrode electrolyzer. Reproduced with permission [289]. Copyright 2023, Academic Press Inc. **g** LSV curves of the OER. **h** LSV curves of the HER. Reproduced with permission [290]. Copyright 2021, WILEY−V C H VERLAG GMBH

The stability of catalyst in seawater electrolyte is very important. Feng et al. uniformly configured (NiFeCoV)S_2_ porous nanosheets on nickel foam [289]. The resulting raw porous nanosheets have a large active surface area and a high number of active sites (Fig. 11a, b). The multiple elements in this catalyst have electronic co-modulation effects, which all contribute to mass transfer and improve catalytic performance. The catalyst achieved excellent electrochemical performance in both alkaline and natural seawater and was even able to withstand durability testing in seawater without hypochlorite precipitation (Fig. 11c−e). When the catalyst was used as anode and cathode to construct a complete water/seawater cracking electrolyzer (Fig. 11f), the cell voltage in alkaline seawater and natural seawater was only 1.69 and 1.77 V at 100 mA cm^−2^, respectively. This phenomenon indicates that the practical application of high-efficiency water/seawater electrolysis is promising. Wu et al. synthesized self-supported Ni_2_P–Fe_2_P. The catalyst has the advantages of fast electron transfer rate, corrosion resistance, good hydrophilicity and excellent activity in seawater electrolysis [290]. The overpotential of Ni_2_P–Fe_2_P in 1 M KOH seawater was 581 mV at 100 mA cm^−2^ (Fig. 11g, h). Liu developed CoP_*x*_@FeOOH catalysts, which was stabilized for 80 h at a high current of approximately 500 mA cm^−2^ [291]. Chang et al. developed a FeP–NiSe_2_ NF catalyst that was stable for more than 8 days in alkaline seawater electrolyte [292].

#### A Solution Containing Small Molecules as Electrolyte

The oxidation-assisted electrolysis of aquatic hydrogen by small molecules greatly reduces the power consumption compared with other electrolytes. The principle is that the degradation of small molecules reduces the voltage of the electrolyzer and produces high value-added value products [293]. At present, some small molecules have been linked to hydrogen production by electrolysis of water, such as urea, hydrazine, aldehyde, alcohol, glycerol, xylose, glucose and plastic upgrading [275, 294–306]. The corresponding properties of various electrocatalysts about small molecule are summarized in Table 3.

**Table 3:** Electrochemical properties of electrocatalysts in small molecule solutions

Type of catalyst	Catalysts	Electrolyte	Anode potential	Cathode potential	Two-electrode setup (V)	Stability	References
Transition metal	Pd/Ni Co_2_O_4_	1 M KOH + 0.5 M N_2_H_4_	*E*_10_ = − 6 mV	*η*_10_ = 294 mV	E_10_ = 0.35	12 h	[307]
	p-VHCF	0.5 M H_2_SO_4_ + 0.1 M N_2_H_4_	–	–	–	6000 cycles	[308]
	Ni_*x*_P/Ni/ NF	0.5 M N_2_H_4_·H_2_O + 1.0 M NaOH	*E*_1215_ = 300 mV	–	*V*_oc_ = 0.94	100 h	[309]
	6W-O-CoP/NF	1 M KOH + 0.1 M N_2_H_4_	*E*_1000_ = 78.99 mV	*η*_1000_ = 185.60 mV	*E*_100_ = 1.634	25 h	[310]
	Ni–Co–P/NF	1 M KOH + 0.1 M N_2_H_4_	*E*_1000_ = 176 mV	*η*_10_ = 37 mV	*E*_500_ = 0.498	100 h	[311]
	NiMo/ Ni_2_P	1 M KOH + 0.5 M N_2_H_4_	*E*_10_ = − 17 mV	*η*_10_ = − 15 mV	*E*_100_ = 0.181	50 h	[312]
	NiMoV LDH	1 M KOH + 0.33 M urea	*E*_100_ = 1.330 V	–	*E*_100_ < 1.63	15 h	[313]
	NiMn_0.22_-LDH	1 M KOH + 0.33 M urea	*E*_100_ = 1.436 V	–	*E*_100_ = 1.436	> 110 h	[314]
	KMO@ NF	1 M KOH + 1 M urea	*E*_10_ = 1.31 V	*η*_20_ = 173 mV	*E*_100_ = 1.697	1000 cycles	[315]
	NiO/CuO@CuM	1 M KOH + 0.5 M urea	*E*_100_ = 1.39 V	–	–	36 h	[316]
	FeP_4_nanotube@Ni–Co–P nanocage	1 M KOH + 0.5 M urea	*E*_10_ = 1.37 V	–	*E*_10_ = 1.52	20 h	[317]
	V–Fe Ni_3_N	1 M KOH + 0.33 M urea	*E*_10_ = 1.382 V	*η*_10_ = 79 mV	*E*_10_ = 1.46	120 h	[318]
	N–Mo–Ni/NF	1 M KOH + 0.1 M benzyl alcohol	*E*_100_ = 1.338 V	–	–	75 min	[319]
	Mo-doped Ni_3_S_2_/NF	1 M KOH + 0.3 M urea	*E*_10_ = 1.33 V	*η*_10_ = 90 mV	*E*_10_ = 1.45	120 h	[320]
	(CoNiCuMnMo) Se/CF	1 M KOH + 0.1 M glycerol	*E*_10_ = 1.2 V	–	*E*_10_ = 0.5	25 h	[321]
	N-CoO_*x*_	1 M KOH + 1 M glycerol	*E*_10_ = 1.31 V	*η*_10_ = 265 mV	*E*_10_ = 1.15	12 h	[322]
Precious metal	Co_9_S_8_/ Ni_3_S_2_	1 M KOH + 50 mM xylose	*E*_780_ = 1.6 V	*η*_100_ = 180 mV	–	3 runs	[323]
	Cu(OH)_2_/Cu foam	1 M KOH + 0.1 M glucose	*E* = 1.0 V	–	*E*_100_ = − 0.92	10 h	[324]
	NiMnO_n_/OCNT	1 M KOH + 1 M glucose	*E*_10_ = 1.3 V	*η*_10_ = 170 mV	*E*_10_ = 1.41	30 h	[325]
	P/Fe–NiSe_2_	1 M KOH + 0.7 M N_2_H_4_	*E*_100_ = 200 mV	*η*_100_ = 168 mV	–	100 h	[326]
	Ni_3_N/ W_5_N_4_	1 M KOH + PET	*E*_10_ = 1.33 V	*η*_10_ = 31 mV	*E*_10_ = 1.4	300 h	[327]
	F-CoP/CF	1 M KOH + 0.2 M N_2_H_4_	*E*_1000_ = 41 mV	*η*_1000_ = 90 mV	*E*_1000_ = 0.49	360 h	[328]
	RhRu_0.5_	1 M KOH + 0.01 M N_2_H_4_	*E*_100_ = 54 mV	–	*E*_100_ = 0.054	80 h	[329]
	MoO_*x*_/Pt	1 M KOH + 0.1 M glycerol	–	*η*_200_ = 63 mV	*E*_10_ = 0.70	—	[330]
	Ru/α-MnO_2_	0.05 M H_2_SO_4_ + 0.5 M N_2_H_4_	*E*_10_ = 0.166 V	–	*E*_10_ = 0.254	1000 h	[331]
	Ru-Cu_2_O/CF	1 M KOH + 0.5 M N_2_H_4_	*E*_10_ = 0.041 V	*η*_10_ = 31 mV	*E*_10_ = 0.0174	18 h	[332]
	PtIr NWs/C	0.5 M H_2_SO_4_ ethanol	–	*η*_10_ = 150 mV	*E*_10_ = 0.61	3000 s	[333]
	Ru-Co_2_P/N–C/NF	1 M KOH + 0.5 M urea	*E*_10_ = 1.351 V	*η*_10_ = 65 mV	*E*_100_ = 1.58	20 h	[334]
	P-mAuRh film/NF	1 M KOH + 0.33 M urea	*E*_50_ = 1.35 V	*η*_100_ = 73 mV	*E*_100_ = 1.47	35 h	[335]
	Ru-(Ni/Fe) C_2_O_4_	1 M KOH + 0.1 M N_2_H_4_	*E*_50_ = 0.079 V	*η*_10_ = 42 mV	*E*_10_ = 0.01	50 h	[336]
	Pt/CoSe_2_	0.5 M H_2_SO_4_ + 1.0 M CH_3_OH	–	*η*_10_ = 29 mV	*E*_10_ = 0.65	1000 cycles	[337]
	CuAg_glv_/ Cu	1 M KOH + 250 mM furfural	*E*_248_ = 0.4 V	–	*E*_100_ = 0.21	5.5 h	[338]
Metal-free	Se@C-1000	1 M KOH + 0.5 M N_2_H_4_	*E*_10_ = 114 mV	–	–	24 h	[339]
	NC/BNC	1 M KOH + 0.1 M N_2_H_4_	*E*_10_ = 479 mV	–	*E*_100_ = 0.90	30 h	[340]

The oxidation of alcohols instead of conventional electrolysis of water can reduce CO_2_ emissions and energy consumption. In response to this, Ma et al. proposed a method of electrolyzing water using ethanol small molecule electrolytes for hydrogen production [341]. Its principle is to generate pure hydrogen in the cathode chamber while oxidizing ethanol on anode, which promotes electrolysis of water. In addition, high value-added product 2, 5-furanediformic acid (FDCA) can be obtained by electrooxidation of 5-hydroxymethylfurfural (HMF). Nawaz et al. prepared CoO and CoO-NiO nanostructures for the selective oxidation of HMF to FDCA [342]. The catalyst achieved complete HMF conversion at 1.38 V RHE with 99% FDCA yield and 99.2% Faradaic efficiency. It was shown that Ni added to CoO–NiO produced a significant effect by reducing the charge transfer resistance and improving the oxidative activity of the electrochemical surface area. Wang et al. prepared CuO–Ni(OH)_2_ heterostructured nanosheets for high-efficiency electrocatalytic oxidation of HMF to produce FDCA [343]. The HMF conversion rate of CuO–Ni(OH)_2_ nanosheets was 100%, the FDCA yield was 99.8% and the Faraday efficiency was 98.4%. The synergistic interaction between CuO and Ni (OH)_2_ is the main reason for the good catalytic activity of the catalyst.

The large amount of plastic waste has become an urgent global problem, and how to deal with this organic waste is receiving increasing scientific and industrial attention. Recently, research on the conversion of plastic waste to hydrogen fuel has been carried out. Wang et al. prepared a nickel-foam-based nitrogen-doped Ni_3_P–NiMoO_4_ heterostructure array catalyst (N–Ni_3_P–NiMoO_4_/NF) for the coupling of electrical recovery and hydrogen production of PET waste plastics [344]. Electron/mass transfer in the electrooxidation of HER and PET hydrolysates is accelerated due to the abundant catalytic active sites provided by the heterogeneous interface structure. N–Ni_3_P–NiMoO_4_/NF can achieve a low hydrogen evolution overpotential of 142 mV at a current density of 100 mA cm^−2^. Xie et al. prepared a 2D metal oxide nanosheet with a porous network for microwax-induced reaction recovery of high-purity hydrogen and carbon nanotubes from waste plastics [345]. The 2D porous structure significantly improves the growth space of the CNTS and enhances the absorption ability, thereby exhibiting a significant H_2_ selectivity of 87.5% and a high H_2_ yield of 60.2 mmol g^−1^ LDPE. Ma et al. fabricated Ni_3_N/W_5_N_4_ Janus nanostructures with a barrier free heterogeneous interface [327]. The Ni_3_N/W_5_N_4_ electrode exhibits Pt-like HER performance and excellent stability (~ 300 h) at industrial currents thanks to interfacial synergy, superhydrophilic surface and multilayer Janus structure. At the same time, Ni_3_N/W_5_N_4_ also showed high activity and selectivity for the electroreforming of plastics, showing a low overpotential of 1.33 V (*η*_10_).

Urea is a cheap, non-toxic and renewable compound, which is commonly used as a fertilizer, and urea oxidation reaction (UOR) has been widely studied in recent years. Lian et al. synthesized Cu(OH)_2_ nanowires with abundant mesopores [346]. This one-dimensional nanowire structure provides an abundance of active sites, resulting in better catalytic kinetics for UOR performance than OER performance. Wu et al. prepared lantern-shaped nanosheets composed of nickel oxide-based porous microspheres and hollow microspheres [347]. This structure improves the charge transfer efficiency of the overall urea cleavage and has excellent electrocatalytic performance. Jiang et al. synthesized a new nanosheet catalyst containing Ni^2+^ and 4-dimethylaminopyridine (Ni–DMAP-t) novel two-dimensional nickel-organic skeleton nanosheets on nickel foam, which showed excellent electrocatalytic activity and strong stability toward the urea oxidation electrolyte [348]. Zhang et al. successfully prepared nickel-doped manganese dioxide (Ni–MnO_2_) nanosheet arrays on nickel foam. The structure of this nanosheet array plus Ni doping changes the electronic structure of Mn atoms, which contributes to the generation of more Mn^3+^ substances with excellent UOR performance [349]. Zequine et al. synthesized CuCo_2_O_4_ nanosheets grown on nickel foam as electrocatalysts, enabling them to be used as efficient UOR electrocatalysts under alkaline conditions [350].

**Fig. 12:**
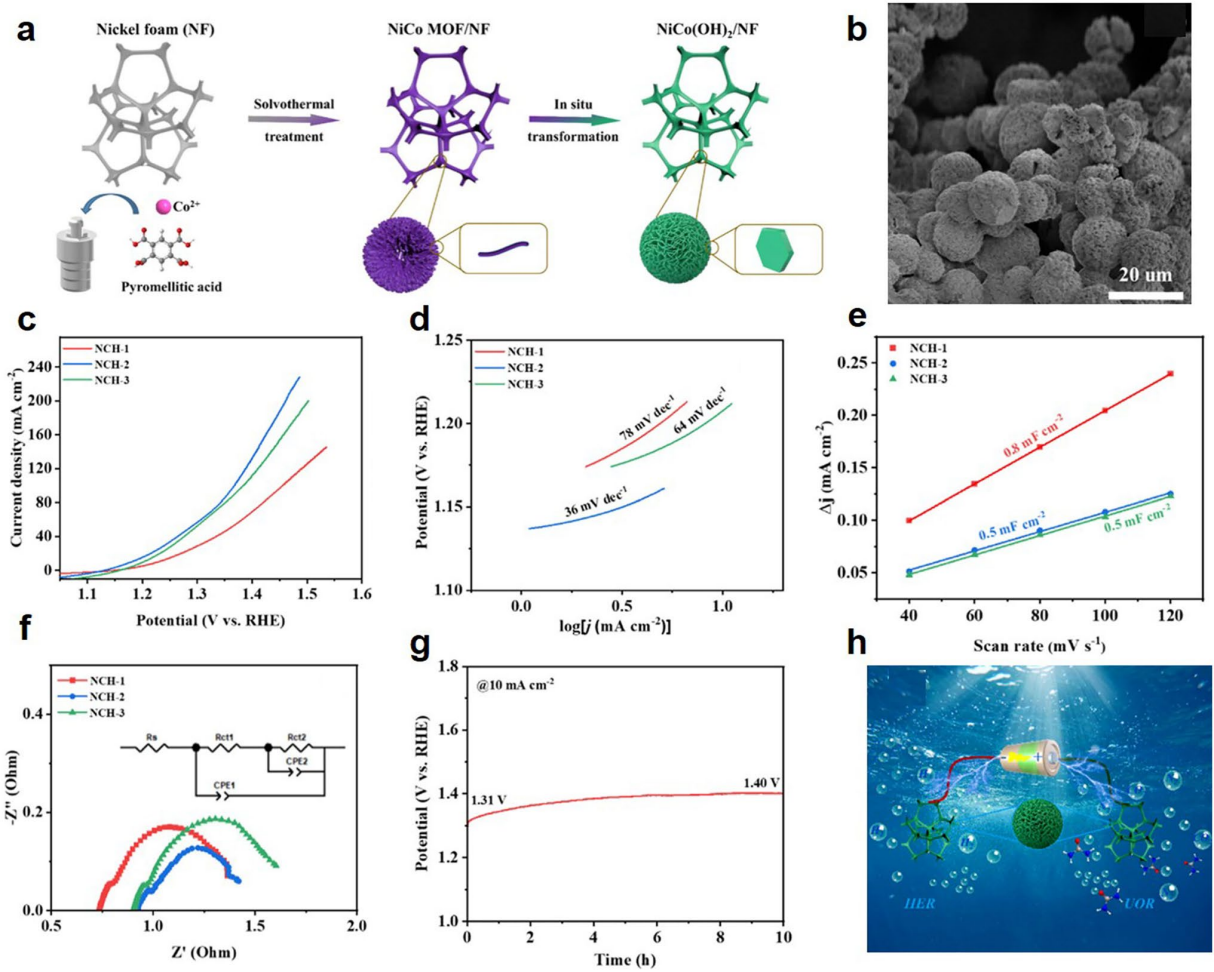
**a** Schematic diagram of sample preparation. **b** SEM image **c** LSV curves of the UOR. **d** Tafel slope. **e** Δ*j* vs. scan rate. **f** EIS plots. **g** Long-term stability test. **h** Schematic diagram of water electrolysis assisted by urea. Reproduced with permission [351]. Copyright 2023, Elsevier BV

Xiang et al. prepared layered microspheres of NiCo(OH)_2_ nanosheets (Fig. 12a, b), which had a spherical structures with rough surfaces. This structure both favors electrolyte penetration and results in an abundant of marginal sites. The intrinsic activity of the catalyst was enhanced by the doping of Co and the combination of in situ generated Ni^3+^ and surface carboxyl groups, resulting in excellent catalytic performance, with a voltage required for urea oxidation being 1.285 V at 50 mA cm^−2^ (Fig. 12c−h) [351].

**Fig. 13:**
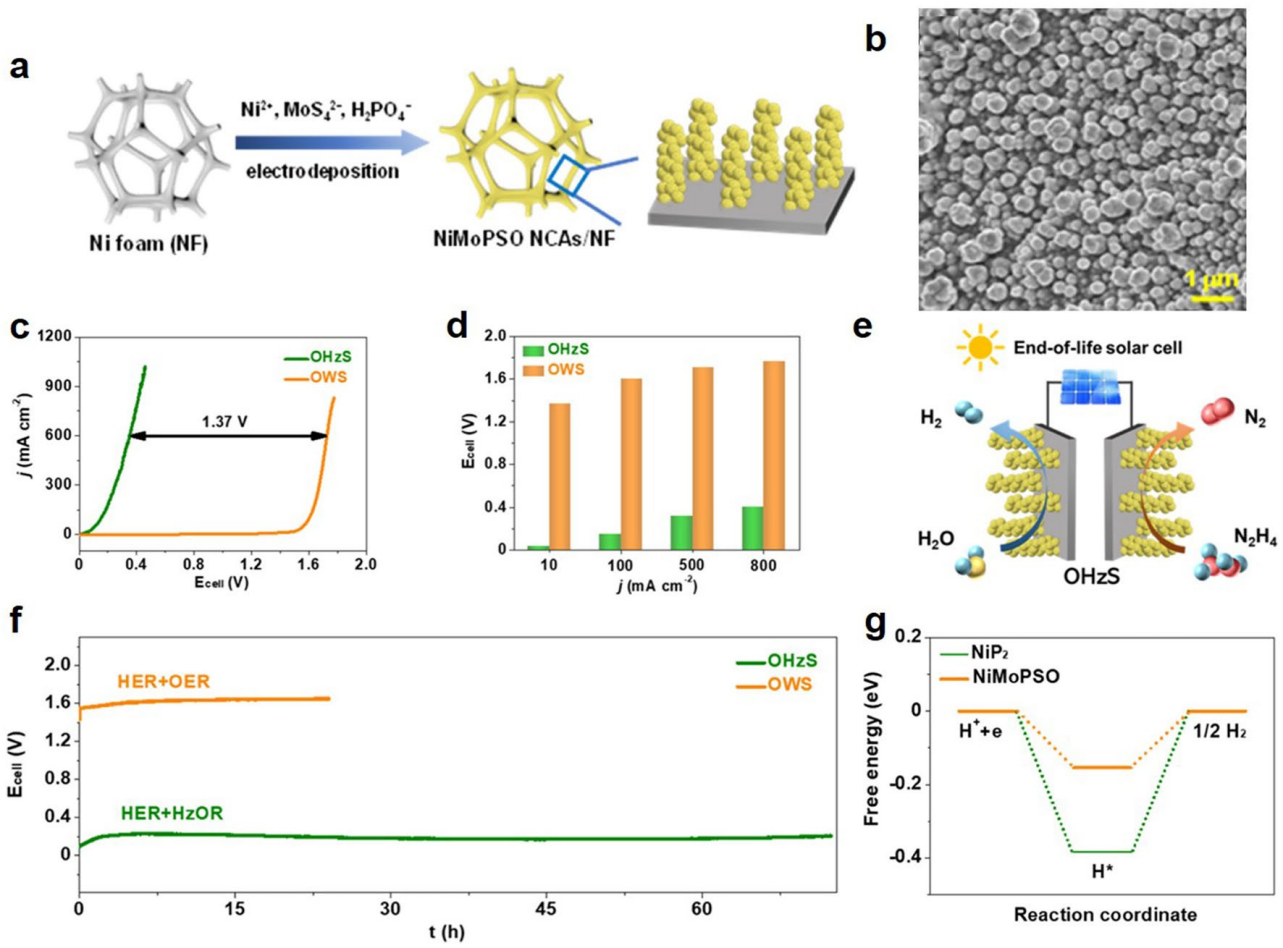
**a** Preparation schematic illustration of NiMoPSO NCAs/NF. **b** SEM image. **c** LSVs cells for OWS and OHzS. **d** Comparison of overpotential. **e** Schematic of solar cell driven OHzS. **f** Long-term stability of hydrazine oxidation. **g** Free−energy profiles of HER on two sample surfaces. Reproduced with permission [352]. Copyright 2022, American Chemical Society

Hydrazine oxidation reaction (HzOR) has the advantage of a low theoretical oxidation potential [305] and stable N_2_ product [329]. Our group reported an entropy-driven highly chaotic nickel-based catalyst coupled to spent solar cells for hydrogen production and hydrazine oxidation [352]. Nickel foam in the form of nanopillars was prepared by electrodeposition (Fig. 13a). SEM image showed that aligned nanorods grew uniformly and densely on the three-dimensional backbone of Ni foam (Fig. 13b). The specific surface area is significantly increased, the gas film caused by rapid bubble accumulation is suppressed and the active sites are reexposed in time. The hydrazine electrolyte solution can generate a high operating current of 1600 mA cm^−2^ at a low voltage of 0.551 V. This solution meets industrial requirements and consumes little energy. It meets industrial requirements with low energy consumption and has excellent bifunctional hydrogen production and hydrazine oxidation performance over 70 h (Fig. 13c−f). Figure 13g shows that the Ni site of NiMoPSO is closer to 0, indicating that the Ni site of NiMoPSO is easier to adsorb and desorb hydrogen, which may account for the better electrocatalytic activity of NiMoPSO in HER.

**Fig. 14:**
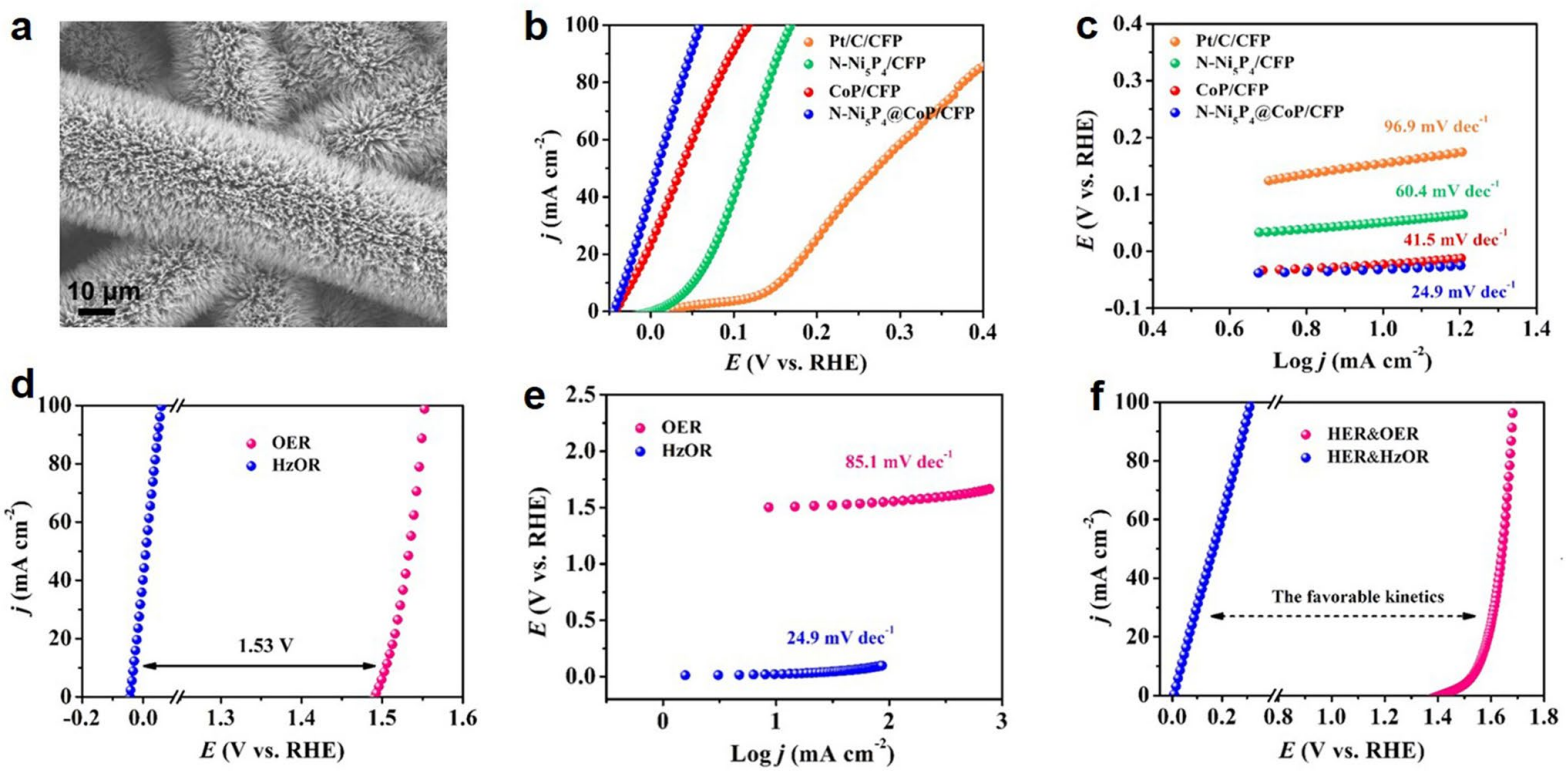
**a** SEM image of N–Ni_5_P_4_@CoP/CFP. **b** LSV curves of the HzOR. **c** Tafel plots. **d** LSV curves, and **e** Tafel slopes of HzOR and OER. **f** LSV curves of N–Ni_5_P_4_ @CoP/CFP for OHzS and OWS electrolyzers. Reproduced with permission [355]. Copyright 2023, Elsevier

Qian et al. achieved a lower potential by coupling MoNi_4_ and NiCo separately [353]. Kim et al. prepared cobalt–manganese bimetallic oxides (Co/MnO) by annealing spinel-structured CoMn_2_O_4_ in a hydrogen atmosphere, and the Co/MnO catalysts showed high activity for the catalytic decomposition of hydrazine [354]. Zhang et al. prepared N–Ni_5_P_4_@CoP/CFP heterogeneous nanowire arrays, which can be observed by scanning electron microscopy to have a dendritic structure (Fig. 14a) [355]. This structure can make the catalyst have more active sites, reduce the gas–solid contact area and accelerate the release of bubbles. Through the redistribution of interfacial charges P and Co into the N–Ni_5_P_4_ and CoP compositions, the nucleophilic P has a higher thermo-neutral H* uptake energy and electrophilic Co has favorable N_2_H_4_* dehydrogenation kinetics, leading to bifunctional catalytic activity toward HER and HzOR, respectively (Fig. 14b−f).

### Economic Analysis of Electrolyzers for Hydrogen Production

The advantage of alkaline electrolysis is that they use the transition metal nickel and its oxide as anodic catalysts. They can be obtained at relatively low cost from a relatively wide range of feedstocks and operate at low temperatures, and they do not require catalysts to activate and produce hydrogen. The cost of the entire alkaline cell is about 1/3–1/5 and as low as 1/7 of the PEM, making the alkaline cell more suitable for industrial applications. The PEM electrolyte resulted in a locally strongly acidic environment due to the large amount of H^+^ produced by the anodic OER. However, catalysts with acidic OER face activity problems due to reaction energy obstacles, and stability problems due to high pressure and strong acid corrosion environment [356]. The noble metals Ru, Ir and their oxides show more excellent catalytic activity and stability in acidic OER reactions, but the limited natural reserves and high cost of Ru and Ir greatly limit their large-scale application [357], resulting in high cost of acidic electrolytic water equipment. In comparison, the use of seawater and small molecule waste liquid is very promising, and the cost is relatively low in theory [358].

### Comparison of Three Electrolyte

**Fig. 15:**
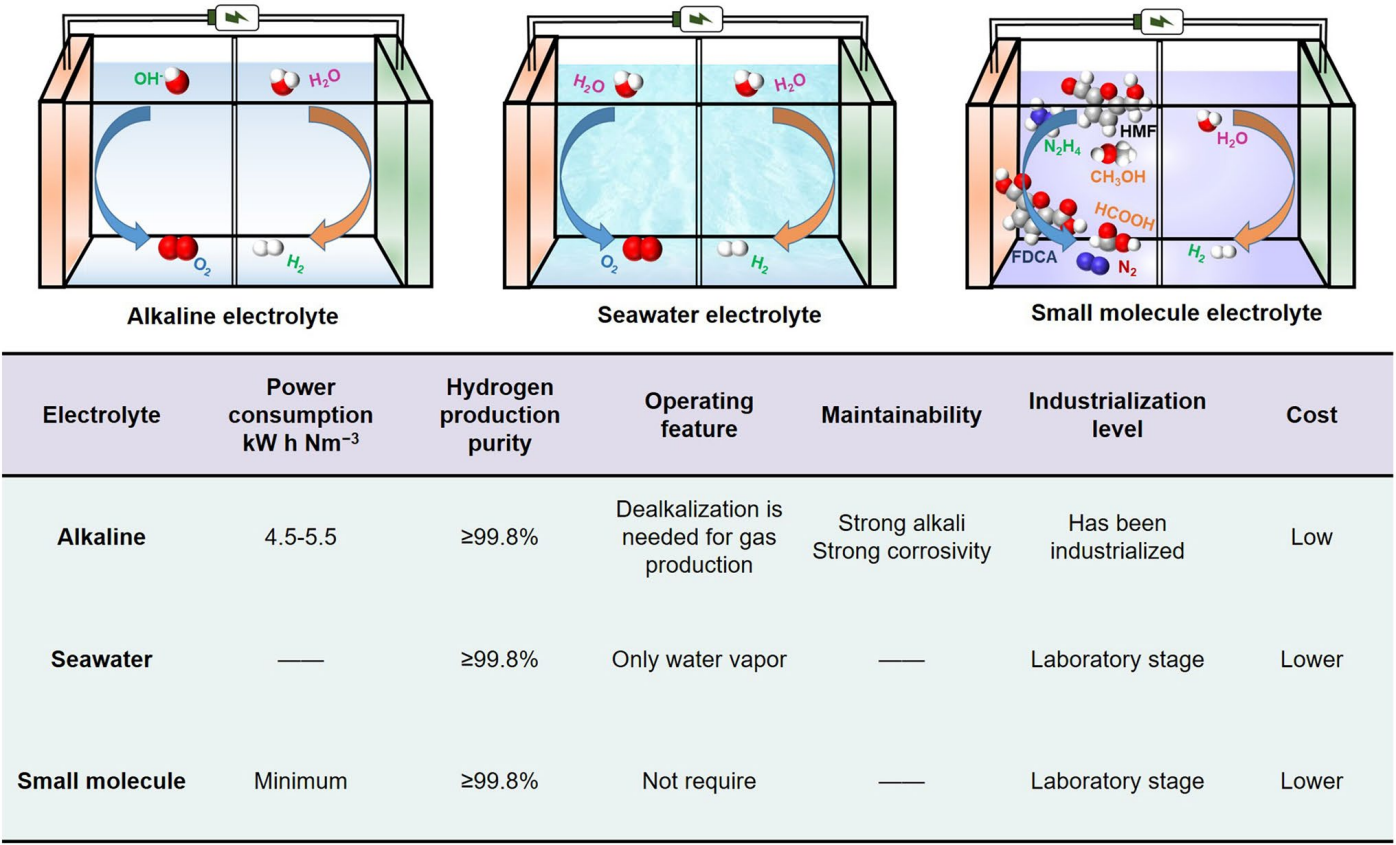
Comparison of different types of electrolytes

By listing the parameters of different electrolytes and comparing their development status horizontally (Fig. 15), we can find that there are still many unresolved issues with the existing technology in terms of electrolyte cost, hydrogen production quality, and equipment lifespan. If these issues are not well balanced, it will be limit the future development of hydrogen production by water electrolysis. Based on these issues, the selection of appropriate electrolyte is an important direction for future electrolyte research.

## Conclusion and Outlook

Since the middle of the twentieth century, there has been a lot of research on hydrogen production by water electrolysis. Hydrogen power is rich in raw materials, widely used, clean and pollution-free, and can emit huge heat. It is a new type of energy that can meet the needs of human survival in the future, and it is very important to improve the hydrogen production technology system. As an important player, green hydrogen is gradually penetrating into the traditional manufacturing field to produce clean and zero-carbon emission products in response to the dual carbon policy. However, the production of hydrogen by electrolysis of water accounts for only about 4 percent of the total global production of hydrogen. In view of the existing problems of water electrolysis hydrogen production technology, in order to better realize the low-cost, high-efficiency and large-scale hydrogen production by water electrolysis, this paper puts forward the following prospects.

### Future Development of Renewable Energy Sources

In view of the environmental pollution caused by the use of traditional energy to generate electricity, hydrogen production from renewable water electrolysis is an emission-free method for hydrogen production. Emissions of greenhouse gases and other pollutants can be minimized during hydrogen production by using solar, wind or other renewable energy sources.

#### Wind Power Generation

Wind energy is a promising renewable energy source that has the potential to reduce greenhouse gas emissions and our dependence on fossil fuels. Wind energy is rapidly increasing and providing an endless supply of eco-friendly electricity. Wind energy is the most important energy technology in the energy industry at present, which can not only change the energy structure, but also save a lot of resources. However, as a natural energy source, the variable rate and instability are the inherent nature of wind energy. Variability is a measure of change from day to day or longer, while instability is random variation due to different weather conditions. Wind power varies from day to day and is also considered to be highly intermittent because its output depends on wind speed, atmospheric conditions and other factors, and this intermittency poses a challenge for grid operators to determine the amount of electricity available at a given moment. For the instability of wind energy, we can adopt a hybrid renewable energy system, synergetic combination of multiple energy sources, such as solar, wind and tide. This hybrid system can generally produce more reliable electricity and is superior to the independent system, improving efficiency and reliability. For example, wind and solar synergies can best mitigate the instability of wind and solar power generation, so we can develop more hybrid renewable energy systems.

#### Tidal Power Generation

Tidal energy has become a competitive and promising renewable energy source due to its high predictability and high energy flow density. Current tidal flow or tidal flow technology is capable of developing and generating renewable energy in many marine environments that exist worldwide. Although tidal current energy is intermittent, it can be predicted in a very accurate manner many years in advance. In other words, electricity providers will be able to easily schedule the integration of tidal energy with standby power ahead of demand. Compared with the traditional power generation mode, it can conserve non-renewable resources, reduce the emission of toxic and harmful substances, has excellent development and utilization potential and value and has high application feasibility. However, tidal power stations have a certain degree of negative impacts on the ecological environment, the most important of which is the destruction of biological habitats, which in turn has a negative impact on the survival and reproduction of many species. Therefore, the survival conditions of coastal fish and birds need to be taken into account when planning tidal power schemes. Although tidal power has not yet been widely used, it has some potential for future power generation. Tides are more predictable than wind and solar power. With the development of science and technology, tidal power generation has great advantages of high efficiency and no pollution. It can be comparable with new energy sources such as solar power generation and wind power generation and is worthy of further development and research.

#### Photovoltaic Power Generation

As one of the most abundant and pure-form renewable energy sources, solar power has the potential to significantly advance the Sustainable Development Goals. Solar energy offers many benefits, including the ability to sustainably meet increasing electricity demand. Solar panels can provide abundant low-carbon energy, thereby contributing to energy security, reducing fossil fuel consumption and emissions and meeting the growing demand for electricity. However, the development of photovoltaic power generation is also affected by many factors. First of all, the cost of photovoltaic materials is relatively high, especially the high conversion rate of photovoltaic single crystal silicon panels, whose production cost is more expensive. This makes the relatively large investment in the construction of PV power systems in some regions and markets a hesitating factor for some potential investors, and solar panels are considered hazardous waste due to their environmental impact and energy loss. Therefore, the production cost of photovoltaic materials should be reduced, the durability and performance of solar cells should be improved, as well as the development of recycling solutions to solve the problem of toxic waste to promote wider photovoltaic applications. Secondly, the stability of photovoltaic power generation system is greatly affected by climatic conditions and geographical location. In some regions, winter weather conditions may lead to a decrease in system performance, thereby affecting generation efficiency. However, for the instability of the PV system, a hybrid wind–solar power generation system can be used, which may be more efficient than a single PV power generation system. For example, in Texas of the USA, wind and solar energy resources are found to be highly complementary, and the combination of wind and solar energy can make the renewable energy production in Texas more reliable and stable. Therefore, more hybrid renewable energy systems can be developed, such as the combination of solar, wind and tide. At the same time, the location of photovoltaic systems is also very important, because the energy production of photovoltaic systems depends on the amount of solar radiation available, and the amount of solar radiation varies in different regions. At present, relevant technologies have been used to locate photovoltaic facilities around the world, so this technology can be improved in the future.

The integration of solar, tidal and wind energy into existing power systems is critical for a sustainable energy future. We can promote the widespread adoption of these sustainable energy sources by addressing the challenges associated with the integration of renewables using advanced forecasting technologies, energy storage technologies, and supportive policies. In addition, by continuing to invest in R&D, more innovation in renewable energy technologies can be stimulated and help achieve their maximum potential in combating climate change and securing a greener future for our planet.

### Future Development of Electrocatalysts

Since the hydrogen evolution and oxygen evolution reactions in the process of water electrolysis are limited by the reaction kinetics, it is crucial to find suitable catalysts. Noble metal electrocatalysts have problems such as high cost, scarcity, and easy poisoning, while transition metal electrocatalysts and nonmetallic catalysts are expected to be potential candidates for hybrid water electrolysis due to their abundant resources and low cost.

#### Noble Metal Catalyst

Noble metal-based electrocatalysts exhibit remarkable catalytic activity and long-term stability in hydrogen evolution reactions due to their excellent hydrogen binding energy. However, limited accessibility and excessive cost of precious metal materials pose barriers to their widespread adoption in industrial Settings. Given the high cost and low natural abundance of precious metals, limiting their consumption is urgently desired. As precious metals are expensive and in limited supply, there is an urgent desire to minimize their consumption. It is therefore possible to shrink the noble metal to a single atom fixed on a porous conductive carbon-based matrix by mixing and alloying it with a less expensive transition metal. Monatomic catalysts have been widely used in electrocatalysis due to their high atom utilization and good electrocatalytic activity. However, due to the high surface energy, increasing the loading of individual atoms would certainly lead to significant accumulation, limiting the catalytic activity. The electronic structure of monatomic catalysts can therefore be fine-tuned by defect engineering, structural modulation and specific alterations to their metal centers and supports.

#### Transition Metal Catalyst

Transition metal catalysts also stand out due to their low cost, abundant reserves and good stability. However, the activity number and conductivity of transition metal catalysts are relatively low, and their performance in electrolyzed water still needs to be further improved. At the same time, the adsorption and desorption characteristics during the reaction are not suitable. However, nonmetallic doping can be regulated in a wide range of directions and applications. Cations in transition metal-based catalysts usually act as true active centers, but the presence of nearby anions can affect the electronic state of the cation, such as the environment and structure, and enhance its inherent catalytic activity.

#### Metal-Free Catalyst

Compared with traditional precious metal or non-precious metal catalysts, nonmetallic catalysts can reduce production costs, achieve energy saving and emission reduction and resource saving. For example, carbon material is a hot research object in nonmetallic catalysts. Carbon materials have the characteristics of high specific surface, good acid and alkali resistance, strong adsorption capacity, thermal stability and chemical stability. However, carbon materials still face many problems in the process of preparation and application. For example, under high temperature conditions, carbon materials are easy to be coked or burned, resulting in inactivation. However, nonmetallic elements can modulate the electronic structure, lattice structure, and surface properties of transition metal catalysts, which may become a new strategy for regulating catalyst activity.

Noble metal catalysts have excellent HER catalytic activity, but due to their storage and price problems, they cannot be used in large-scale industrial applications; therefore, the current research goal is to minimize the loading of noble metals in the catalyst. Transition metal catalysts have the advantages of low cost, simple preparation method, and diverse structure and composition, which have become the focus of current research. Nonmetallic catalysts are mainly carbon materials, which have the advantages of excellent electrical conductivity, strong acid and alkali corrosion resistance, and adjustable structure. The catalytic activity of carbon materials can be improved to a certain extent by doping or manufacturing defects.

### Future Development of Electrolytes

In view of the problems of high cost, poor quality and short equipment life of hydrogen production by electrolytic water, electrolytes with low corrosion, low price and low energy consumption can be selected.

#### Alkaline Solution

Although PEM technology is highly efficient, it relies on precious metals such as platinum and iridium as catalysts, so the high price has become the main factor restricting the further large-scale promotion of PEM. Due to the high temperature problem, SOEC technology faces the problems of high temperature sealing, start-stop rate and impact resistance of ceramic materials. Therefore, the high cost of maintenance has become the main factor restricting the further large-scale promotion of SOEC. AEM technology has attracted wide attention due to its advantages of high efficiency and low cost as the development direction of the next-generation technology. It can use cheaper catalysts such as nickel base on the basis of achieving equivalent or even higher electrolytic efficiency than PEM technology and SOEC technology, and greatly reduce the cost of the whole tank. However, the current anion exchange membrane cannot take into account both efficiency and life, so the breakthrough point of future AEM technology is to develop high stability and long life anion exchange membrane. The research and development of commercial anion-exchange membrane materials is actually to seek the balance between electrolytic efficiency and lifetime. Thinner anion-exchange membranes are fabricated to reduce the dielectric resistivity. Thin membranes are prone to degradation in the local strong alkaline environment generated by electrolytic water, resulting in membrane perforation, which poses a higher challenge to the mechanical stability and life of the exchange membrane material itself. The number and position of functional groups affect the ionic conductivity, which affects the final electrolysis efficiency. At present, aromatic polymers are mainly used as skeleton materials, but their stability is not good. Therefore, constructing ion channels and optimizing ion channel structure are effective strategies to improve the conductivity of ion exchange membranes in the future. By synthesizing polymers with block, graft, and comb structures, it is possible to construct microphase separation structures in AEM, improve the conductivity of hydroxide, extend the service life and develop anion exchange membranes with better comprehensive performance.

#### Seawater

At present, electrolyzed water is mainly freshwater, which will accelerate the shortage of freshwater resources on the earth. Seawater is not limited by borders and territory, and is an inexhaustible resource. However, a major difficulty in hydrogen production from seawater is that the element composition in seawater is complex, which easily leads to catalyst failure. Therefore, it is necessary to develop and synthesize low-cost catalysts with high HER efficiency, high catalytic and structural stability, high impurity tolerance and high selectivity. Achieving industrial-scale hydrogen production is the ultimate goal we pursue, so it is particularly important to prepare efficient and stable catalysts for seawater electrolysis at high current densities. Therefore, improving the interface coupling between catalyst and support is an important contribution to the development of efficient and stable catalysts for hydrogen production from industrial seawater. Moreover, offshore wind power and photovoltaic technology are expected to become the mainstay of future green energy sources due to their advantages of abundant resources and great prospects. Offshore wind power has the advantages of higher wind speed, shorter silent period, and saving land resources from considering noise and other pollution. However, there are some problems such as high construction cost, low energy utilization rate, and difficult transportation. Solar energy resources are abundant in coastal areas, and there is no shielding on the sea surface, which can make full use of water reflected light to improve power generation. Compared with terrestrial photovoltaic, it can be increased by 5%–10%, but there are problems such as high investment cost and environmental impact. Therefore, the breakthrough point of seawater hydrogen production, offshore wind power and offshore photovoltaic should be based on technological innovation, and should be combined with the future energy development trend.

#### Small Molecular Solution

Small molecular solution. Solutions containing small molecules are relatively cheap and abundant, such as urea, hydrazine, alcohol, HMF, formate, ammonia and plastic upgrades. Urea is widely present in agricultural wastewater and the excreta of natural organisms. Therefore, combining the ureaization reaction with electrolytic water can effectively produce H_2_ and purify the urea-rich wastewater, thereby achieving ecological protection and producing clean energy. Hydrazine hydrate is an important chemical raw material and has important applications in industrial production and aerospace fields. Similar to urea, as a chemical with high nitrogen content, it is also easy to cause some damage to the environment. As an important biomass platform molecule, HMF and its product FDCA are promising biomass feedstocks for the production of various downstream chemicals. Therefore, coupling electrocatalytic HMF oxidation with cathodic reduction can improve energy utilization efficiency and economic benefit. Plastic is a kind of toxic waste that is not easy to degrade, so upgrading waste plastic into hydrogen fuel has high environmental and economic benefits. Electrochemical plastic reforming is expected to provide a green, convenient and effective way to recycle plastics. On the one hand, electricity can be obtained from renewable sources and the reaction conditions are relatively mild. On the other hand, value-added chemicals from plastic upgrading on the anode can be combined with hydrogen production on the anode. The required catalysts are required to efficiently drive the HER process and meet the requirements for high activity and selectivity for plastic upgrades. Therefore, industrial wastewater and domestic sewage are used to replace freshwater electrolytes, which will promote the better development of renewable electrolytic systems. However, the problem with small molecule solutions is that some small molecules have poor electrochemical activity. Therefore, the focus of future development should be the designation of electrocatalysts for specific molecules.

**Fig. 16:**
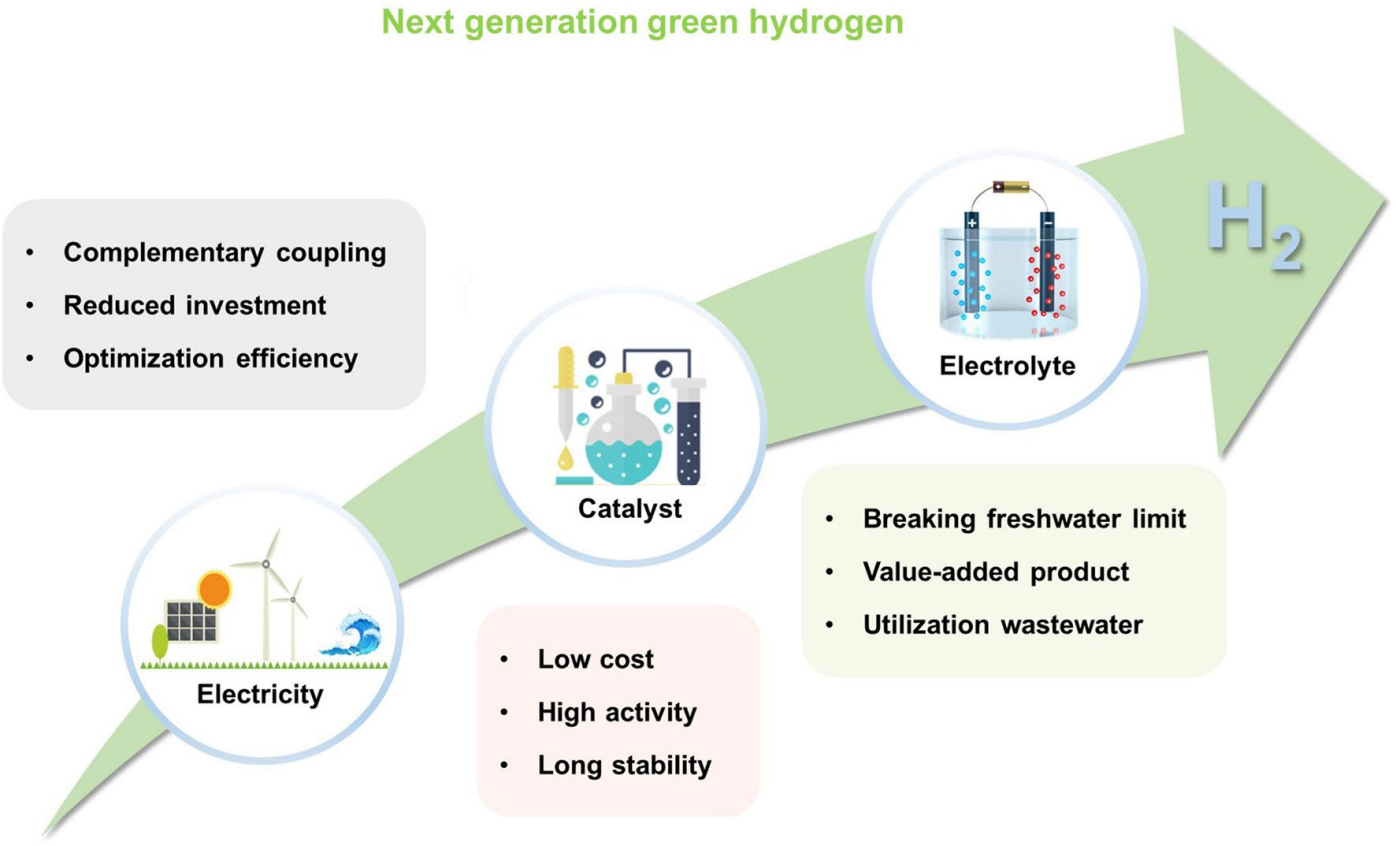
Prospects for future research directions of hydrogen generation from electrolytic water splitting

This review provides an overview of recent advances and notable achievements in innovative strategies for green hydrogen energy through electrochemical processes. Overall, in the development of green energy and the hydrogen economy, renewable water electrolysis to hydrogen technology plays an important role. Development of renewable electricity, design of catalyst structure, and improvement of electrolyzers will work together to break through the cost bottleneck of electrolysis hydrogen production technology. Future research will focus on improving technical efficiency, reducing costs, solving engineering challenges in real-world applications, and advancing the commercialization and widespread adoption of renewable hydrogen (Fig. 16). As more scientific research and innovation work is carried out, new breakthroughs will be made in this field. This cutting-edge review provides a reference for the follow-up research of next water electrolysis hydrogen production technology.
